# Six new polyphenolic metabolites isolated from the *Suillus granulatus* and their cytotoxicity against HepG2 cells

**DOI:** 10.3389/fnut.2024.1390256

**Published:** 2024-04-24

**Authors:** Hanyu Zhao, Miaomiao Xiong, Xiaomin Yang, Lan Yao, Zeyan Wang, Li-an Wang, Zhuang Li, Jinxiu Zhang, Jianhua Lv

**Affiliations:** ^1^College of Life Sciences, Hebei Normal University, Shijiazhuang, China; ^2^Institute of Biology, Hebei Academy of Science, Shijiazhuang, China; ^3^College of Civil Engineering and Architecture, North China Institute of Aerospace Engineering, Langfang, China; ^4^Hebei Collaborative Innovation Center for Eco-Environment, Hebei Normal University, Shijiazhuang, China

**Keywords:** *Suillus granulatus*, phenolic metabolites, mushrooms, macro fungi, cytotoxicity

## Abstract

Edible mushrooms are an important source of nutraceuticals and for the discovery of bioactive metabolites as pharmaceuticals. In this work, six new polyphenolic metabolites suillusol A-D (**1**–**4**), suillusinoic acid (**5**), ethyl suillusinoate (**6**), were isolated from the *Suillus granulatus*. The structures of new compounds were elucidated using high-resolution electrospray ionization mass spectroscopy, nuclear magnetic resonance data, and single-crystal X-ray diffraction analysis. As far as we know, compound **1** represents an unprecedented type of natural product and compound **3** represents a new type of polyphenol fungal pigment, which may be biosynthetically related to thelephoric acid. The cytotoxicity against HepG2 cells of the new compounds were also evaluated. Compound 2 demonstrate significant inhibitory activity against HepG2 cells with IC_50_ values of 10.85 μM, surpassing that of positive control cisplatin. Moreover, compound **1** and **3** also exhibited moderate cytotoxic activity with their IC_50_ values measured at 35.60 and 32.62 μM, respectively. Our results indicate that *S. granulatus* is a rich source of chemical constituents that may provide new lead compounds for the development of anticancer agents.

## Introduction

The genus *Suillus*, a type of ectomycorrhizal fungi known for its high host specificity and commonly found in symbiosis with trees such as pines and firs, is classified within the Boletales order of the Basidiomycetes class (1). In some regions of China, it is commonly referred to as “pine mushrooms.” The *Suillus* genus has attracted growing research interest due to its nutritional value, capacity to produce biologically active secondary metabolites, and potential applications. Since the 1960s, studies have been initiated on the extracts, chemical constituents, and biological activities of this genus (2, 3). Studies have shown that the extracts derived from *Suillus* genus exhibit notable antioxidant, antineoplastic, antimicrobial properties and others (4–13). This implies that they integrate culinary and medicinal properties, being a valuable forest resource with significant economic and ecological benefits (14).

*Suillus granulatus*, a species of edible fungus from the genus *Suillus*, is widely distributed around the world (15). Current investigations into the chemical composition of *S. granulates* have been primarily confined to the analysis of polyprenylphenols and fatty acids, with the notable absence of studies on the complex phenolic metabolites (16–20).

Liver cancer is a malignant tumor with a global distribution, and it is estimated that mortality rate attributed to liver cancer will surpass one million by the year 2030 (21–23). Chemotherapy is a pivotal method in the treatment of liver cancer, especially for patients who are not suitable candidates for surgical removal of the tumor. Nevertheless, given the protracted nature of such regimens, associated adverse reactions, and the propensity for chemoresistance development, the therapeutic effectiveness of chemotherapy often falls short of expectations (24, 25). Therefore, natural products with a reliable anti-hepatocarcinoma effect that are less toxic and have fewer side effects have received increasing attention.

To identify new natural products with anti-hepatocarcinoma activity, we investigated chemical constituents of *S. granulatus* and isolated six novel polyphenolic compounds Subsequently, these compounds were evaluated for their cytostatic potential against hepatoma cell lines HepG2. Remarkably, all six compounds exhibited varying degrees of cytotoxic effects, with compound **2** exhibiting notably higher activity compared to the positive control cisplatin. The present study delineates the isolation, structural characterization, and antitumor activity against HepG2 cells of these novel polyphenolic compounds.

## Materials and methods

### General experimental procedure

HR-ESI-MS spectra were acquired on a Waters Xevo G2 Q-TOF mass spectrometer (Waters Co., Milford, MA, USA). NMR spectra were recorded on n a Bruker AM-600 spectrometer with TMS as an internal standard (Bruker, Ettlingen, German). NMR spectra were recorded at 25°C on a Bruker AM-600 spectrometer equipped with a cryoprobe, and deuterated solvents signal was used as an internal standard. The column chromatography (CC) was performed on YMC RP-18 gel (Fuji Silysia Chemical Ltd., Kasugai, Japan) and silica gel (200–300 mesh, Qingdao Marine Chemical Ltd., Qingdao, China). High-performance liquid chro-matography (HPLC) was performed on Waters 2,535 chromatography system (Waters, Milford, MA, USA) equipped with a Waters 2,489 UV/visible detector with a YMC-Pack ODS-A (250 × 10 mm, 5 μm) column (YMC Co., Ltd., Kyoto, Japan).

### Mushroom material

The fresh fruiting bodies of *S. granulatus* were collected from the pine forests in Maojinba National Forest Park in Longhua Country, Chengde City, Hebei Province, China, in September 2022. It was morphologically identified by Prof. Li-an Wang (Hebei Normal University, China), and then verified by molecular analysis.

### Extraction and isolation

The fruiting bodies of *S. granulatus* were subjected to air-drying for 48 h (dry weight, 20.5 kg) followed by extraction using 95% ethanol (60 L, three times) for 24 h to obtain the crude extract weighing 3,025 g. The crude extract was suspended in 3 L of water and then sequentially partitioned with petroleum ether (2 × 3 L, 2 h each), ethyl acetate (3 × 3 L, 2 h each), and n-butanol (2 × 3 L, 2 h each).

The petroleum ether fraction (PE, 137 g) was separated into eleven fractions (PE1–PE11) using silica gel CC eluted with petroleum ether/ethyl acetate (100:1–1:1). PE2 (1.4 g) was further purified by semi-preparative RP-HPLC (YMC ODS-A column, 250 × 10 mm, 5 μm, 2.5 mL/min, acetonitrile/water, 95:5) to obtain compound **5** (8 mg, *t*_R_ = 32 min).

The ethyl acetate fraction (EA, 380 g) was separated into eight fractions (EA1–EA8) using silica gel CC eluted with dichloromethane/methanol (100:1–1:1). EA4 (31 g) and EA5 (43 g) were pooled and further purified by ODS reversed-phase silica gel CC eluting with a methanol/water (1:9–8:2) gradient system, resulting in the isolation of six sub-fractions designated as EA4A-EA4F. From EA4D (6 g), compounds **1** (acetonitrile/water, 25:75, 4 mg, *t*_R_ = 20.5 min), **2** (acetonitrile/water, 30:70, 6 mg, *t*_R_ = 13.3 min), **4** (acetonitrile/water, 30:70, 6 mg, *t*_R_ = 29.7 min), and **6** (acetonitrile/water, 25:75, 16 mg, *t*_R_ = 27.1 min) were isolated by semi-preparative RP-HPLC (2.5 mL/min).

The n-butanol fraction (54 g) was separated by ODS reversed-phase silica gel CC eluting with a methanol/water (1:9–8:2) gradient system, resulting in the isolation of six fractions designated as BA1–BA6. Compound **3** was separated as crystals from the BA6 fraction.

*Suillusol A (**1**)*: light yellow powder; [α]${\;}_{\text{D}}^{25}$ 0 (*c* 0.1, MeOH); UV (MeOH) λ_max_ 285, 375 nm; IR_max_ 3,319, 1,739, 1,615, 1,515, 1,460, 1,438, 1,384, 1,317, 1,260, 1,173, 1,124 cm^−1, 1^H and ^13^C NMR data see Table 1; HR-ESI-MS (*m/z* 311.0555 [M–H]^−^, calcd. 311.0561).

**Table 1:** The ^1^H and ^13^C NMR data for **1** in CD_3_OD.

**No**.	**^1^H NMR**	**^13^C NMR**
2	6.00 (1H, d, 2.0)	75.6 (d)
3	—	121.5 (s)
4	—	155.7 (s)
5	6.69 (1H, d, 2.8)	111.2 (d)
6	—	153.1 (s)
7	6.80 (1H, dd, 8.8, 2.8)	121.7 (d)
8	6.74 (1H, d, 8.8)	119.0 (d)
9	—	148.2 (s)
10	—	117.6 (s)
11	—	173.3 (s)
12	5.21 (1H, dd, 17.3, 2.0) 5.39 (1H, d, 17.3)	70.1 (t)
1′	—	131.7 (s)
2′	6.77 (1H, d, 1.8)	115.1 (d)
3′	—	146.5 (s)
4′	—	147.2 (s)
5′	6.69 (1H, d, 8.2)	116.2 (d)
6′	6.67 (1H, dd, 8.2, 1.8)	119.7 (d)

*Suillusol B (**2**)*: light yellow powder; UV (MeOH) λ_max_ 289, 347 nm; IR_max_ 3,305, 1,685, 1,612, 1,589, 1,509, 1,438, 1,383, 1,235, 1,182, 1,117 cm^−1, 1^H and ^13^C NMR data see Table 2; HR-ESI-MS (*m/z* 299.0555 [M–H]^−^, calcd. 299.0561).

**Table 2:** The ^1^H and ^13^C NMR data for **2** in CD_3_OD.

**No**.	**^1^H NMR**	**^13^C NMR**
2	—	154.2 (s)
3	—	138.9 (s)
4	—	174.4 (s)
5	7.38 (1H, d, 2.8)	108.1 (d)
6	—	155.7 (s)
7	7.16 (1H, dd, 9.1, 2.8)	124.1 (d)
8	7.37 (1H, d, 9.1)	120.5 (d)
9	—	151.1 (s)
10	—	124.1 (s)
1′	—	129.5 (s)
2′	6.77 (1H, d, 1.6)	116.9 (d)
3′	—	146.4 (s)
4′	—	145.3 (s)
5′	6.68 (1H, d, 8.1)	116.5 (d)
6′	6.65 (1H, dd, 8.1, 1.6)	121.3 (d)
7′	3.97 (2H, s)	35.1 (t)

*Suillusol C (**3**)*: yellow crystals (methanol); UV (MeOH) λ_max_ 291, 377 nm; IR_max_ 3,413, 1,691, 1,510, 1,438, 1,376, 1,347, 1,251, 1,174, 1,084 cm^−1, 1^H and ^13^C NMR data see Table 3; HR-ESI-MS (*m/z* 353.0295 [M–H]^−^, calcd. 353.0303).

**Table 3:** The ^1^H and ^13^C NMR spectral data for **3** in DMSO-*d*_6_.

**No**.	**^1^H NMR**	**^13^C NMR**
1	—	172.0 (s)
2	—	142.6 (s)^*^
3	—	146.1 (s)
4a	—	142.5 (s)^*^
5	—	154.7 (s)
6a	—	142.9 (s)
7	7.32 (1H, d, 8.9)	117.0 (d)
8	7.01 (1H, dd, 8.9, 2.9)	117.8 (d)
9	—	154.2 (s)
10	8.75 (1H, d, 2.9)	111.6 (d)
10a	—	116.3 (s)
10b	—	120.2 (s)
1′	—	121.1 (s)
2′	7.72 (1H, d, 2.2)	114.6 (d)
3′	—	145.3 (s)
4′	—	148.0 (s)
5′	6.93 (1H, d, 8.5)	115.7 (d)
6′	7.61 (1H, dd, 8.5, 2.2)	119.9 (d)
2-OH	10.06 (1H, brs)	—
9-OH	9.82 (1H, brs)	—
3′-OH	9.48 (1H, brs)	—
4′-OH	9.67 (1H, brs)	—

^*^Interchangeable.

*Suillusol D (**4**)*: yellow powder; UV (MeOH) λ_max_ 271, 385 nm; IR_max_ 3,380, 1,735, 1,655, 1,616, 1,512, 1,465, 1,265 cm^−1, 1^H and ^13^C NMR data see Table 4; HR-ESI-MS (*m/z* 311.0542 [M+H]^+^, calcd. 311.0550).

**Table 4:** The ^1^H and ^13^C NMR spectral data for **4** in DMSO-*d*_6_.

**No**.	**^1^H NMR**	**^13^C NMR**
2	—	166.6 (s)
3	—	94.3 (s)
3a	—	155.5 (s)
4a	—	144.3 (s)
5	7.03 (1H, s)	103.6 (d)
6	—	148.1 (s)
7	—	143.7 (s)
8	7.00 (1H, s)	112.8 (d)
8a	—	111.7 (s)
9	7.22 (1H, s)	106.4 (d)
9a	—	138.9 (s)
1′	—	120.1 (s)
2′, 6′	7.92 (2H, d, 8.7)	127.4 (d)
3′, 5′	6.89 (2H, d, 8.7)	115.6 (d)
4′	—	156.6 (s)

*Suillusinoic acid (**5**)*: light yellow powder; UV (MeOH) λ_max_ 311, 368 nm; IR_max_ 3,296, 1,716, 1,684, 1,608, 1,508, 1,458, 1,383, 1,237, 1,174 cm^−1, 1^H and ^13^C NMR data see Table 5; HR-ESI-MS (*m/z* 355.0458 [M–H]^−^, calcd. 355.0459).

**Table 5:** The ^1^H and ^13^C NMR data for 5 and 6 in CD_3_OD.

**No**.	**5**		**6**	
	^1^ **H NMR**	^13^ **C NMR**	^1^ **H NMR**	^13^ **C NMR**
1	—	200.2 (s)	—	197.9 (s)
2	—	150.3 (s)	—	150.9 (s)
3	—	140.5 (s)	—	137.4 (s)
3a	—	93.7 (s)	—	91.9 (s)
4a	—	154.6 (s)	—	153.8 (s)
5	6.65 (1H, d, 8.6)	111.7 (d)	6.69 (1H, d, 8.7)	111.7 (d)
6	6.58 (1H, dd, 8.6, 2.2)	116.8 (d)	6.64 (1H, brdd, 8.7, 2.4)	117.4 (d)
7	—	152.6 (s)	—	153.4 (s)
8	6.79 (1H, d, 2.2)	112.1 (d)	6.82 (1H, brd, 2.4)	112.2 (d)
8a	—	124.8 (s)	—	123.8 (s)
8b	3.94 (1H, brs)	57.7 (d)	4.12 (1H, brs)	56.9 (d)
9	—	178.1 (s)	—	172.9 (s)
1′	—	126.3 (s)	—	125.3 (s)
2′	7.77 (1H, brs)	117.7 (d)	7.65 (1H, d, 2.1)	117.2 (d)
3′	—	145.7 (s)	—	146.1 (s)
4′	—	147.8 (s)	—	148.3 (s)
5′	6.77 (1H, d, 8.4)	115.9 (d)	6.79 (1H, d, 8.4)	116.1 (d)
6′	7.62 (1H, brd, 8.4)	123.6 (d)	7.43 (1H, dd, 8.4, 2.1)	122.8 (d)
1^′′^	—	—	4.16–4.22 (2H, m)	63.4 (t)
2^′′^	—	—	1.12 (3H, t, 7.1)	14.2 (q)

*Ethyl suillusinoate (**6**)*: light yellow powder; UV (MeOH) λ_max_ 289, 370 nm; IR_max_ 3,295, 1,711, 1,685, 1,609, 1,509, 1,439, 1,384, 1,239, 1,175 cm^−1, 1^H and ^13^C NMR data see Table 5; HR-ESI-MS (*m/z* 383.0762 [M–H]^−^, calcd. 383.0772).

### Cytotoxic activity

Cell viability assay was determined by the Cell Counting Kit-8 Assay Kit (CCK8, Bioss, China). The experiment was determined based on the method of Liu et al. (26). HepG2 cells (100 μL, 5 × 10^4^/mL) were seeded onto a 96-well plate overnight. The cells were treated with different concentrations (5, 10, 20, 40, 80, 160 μM) of samples (100 μL) dissolved in medium for 72 h at 37°C and 5% CO_2_. Then, 10 μL CCK-8 solution was added to each well, and the plate was protected from light and incubated at 37°C for 1 h. Afterwards, the optical density was measured by a microplate reader at 450 nm. Each experiment was repeated three times. Cisplatin was used as a positive control, and the above operation was repeated.

## Results and discussion

### Structure elucidation

**Figure 1:**
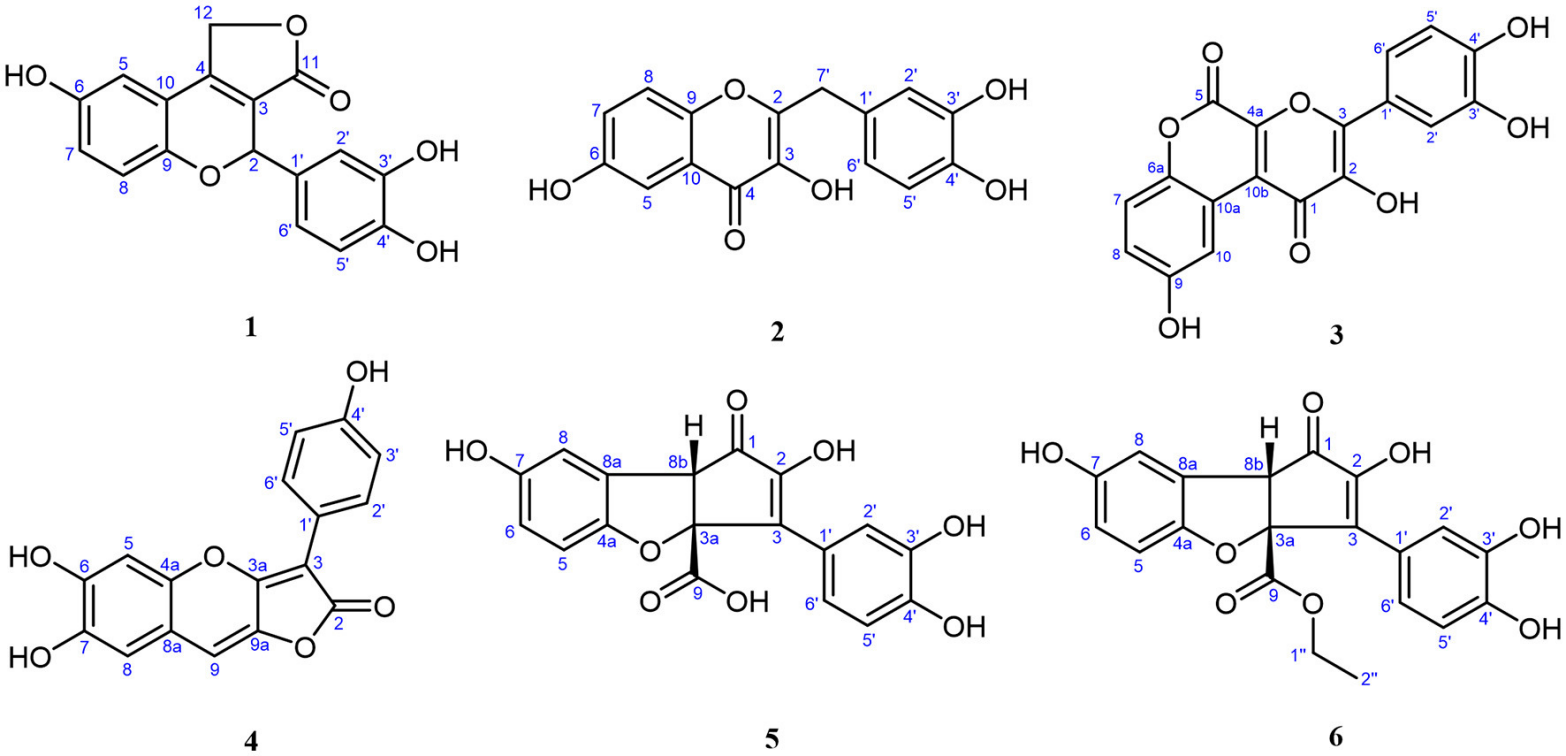
Structures of compounds **1**–**6**.

**Figure 2:**
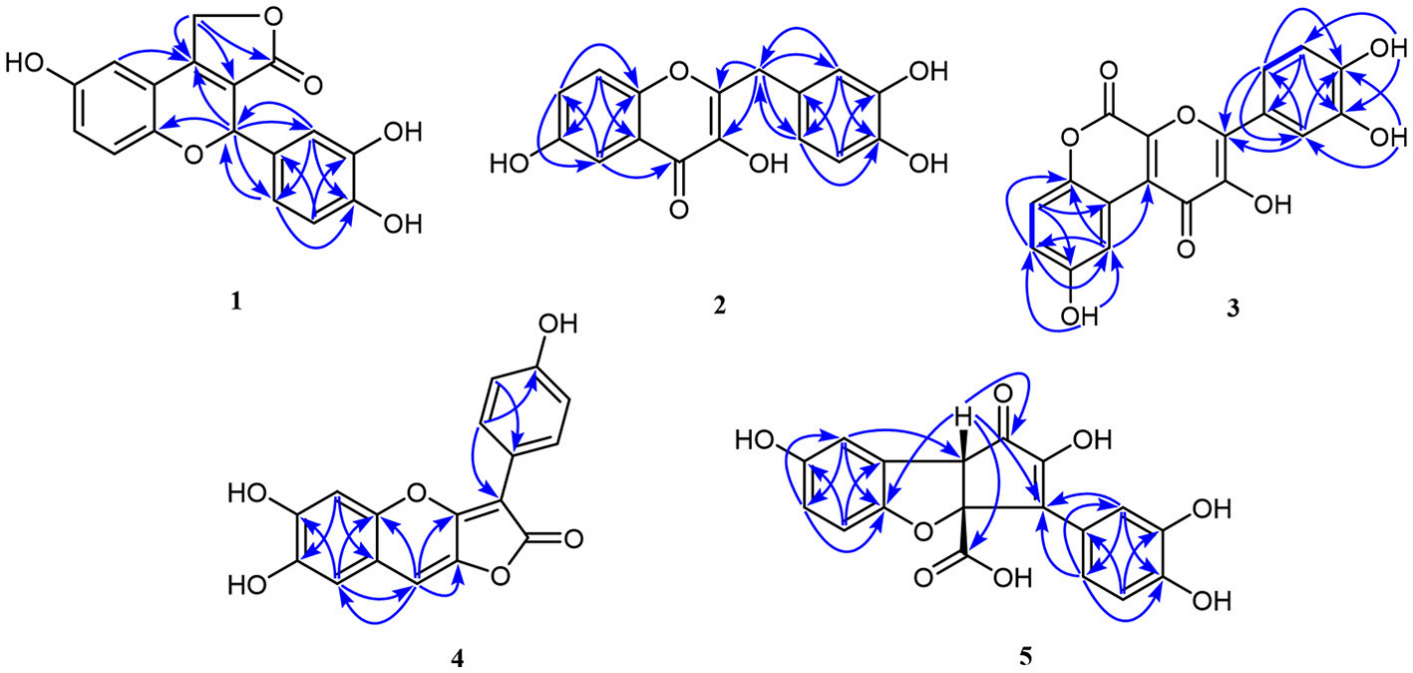
The HMBC correlations of compounds **1**–**5**.

Compound **1**, light yellow powder, possessed a molecular formula of C_17_H_12_O_6_ by the negative HR-ESI-MS (*m/z* 311.0555 [M–H]^−^, calcd. 311.0561), accounting for 12 degrees of unsaturation. The ^1^H NMR spectrum (Table 1) in CD_3_OD of **1** showed two sets of characteristic 1,3,4-trisubstituted benzene ring signals at δ_H_ 6.80 (1H, dd, *J* = 8.8, 2.8 Hz, H-7), 6.74 (1H, d, *J* = 8.8 Hz, H-8), 6.69 (1H, d, *J* = 2.8 Hz, H-5), 6.77 (1H, d, *J* = 1.8 Hz, H-2′), 6.69 (1H, d, *J* = 8.2 Hz, H-5′), and 6.67 (1H, dd, *J* = 8.2, 1.8 Hz, H-6′), one downfield oxygen-bearing methine proton at δ_H_ 6.00 (1H, d, *J* = 2.0 Hz, H-2), and one downfield oxygenated methylene signal at δ_H_ 5.21 (1H, dd, *J* = 17.3, 2.0 Hz, H-12a) and 5.39 (1H, d, *J* = 17.3 Hz, H-12b), which hinted the existence of three phenolic hydroxy protons. The ^13^C NMR spectrum (Table 1) showed a total of 17 carbon resonances, including one lactone carbonyl carbon at δ_C_ 173.3 (s, C-11), 14 aromatic or olefinic carbons due to two benzene rings and a double bond group, as well as two oxygenated carbons at δ_C_ 75.6 (d, C-2) and 70.1 (t, C-12). The analysis of the degrees of unsaturation suggested that there were two aliphatic rings in **1**. A detailed analysis of the HMBC correlations (Figure 2) allowed to infer the presence of a 3,4-dihydroxyphenyl moiety, which was attached to the oxygenated methine carbon at δ_C_ 75.6 (d, C-2). While the other 1,3,4-trisubstituted benzene ring was located on a chromene nucleus by the observable HMBC correlations from H-5 [δ_H_ 6.69 (1H, d, *J* = 2.8 Hz)] and H-2 [δ_H_ 6.00 (1H, d, *J* = 2.0 Hz] to C-4 [δ_C_ 155.7 (s)] and C-9 [δ_C_ 148.2 (s)]. Furthermore, the ^4^*J* coupling constant of H-5 indicated a phenolic hydroxy group at C-6, which was confirmed by analysis of the HMBC correlations (Figure 2). By consideration of the NMR shifts of the two unsolved carbons (C-11 and C-12) and the degrees of unsaturation, the other aliphatic ring system was assigned to a γ-lactone ring, which was confirmed by the HMBC correlations from H_2_-12 [δ_H_ 5.21 (1H, dd, *J* = 17.3, 2.0 Hz) and 5.39 (1H, d, *J* = 17.3 Hz)] to C-3 [δ_C_ 121.5 (s)], C-4 [δ_C_ 155.7 (s)] and C-11 [δ_C_ 173.3 (s)]. The lack of significant optical rotation observed for compound **1**, indicated that the compound may in fact be present as racemic mixtures. Therefore, the structure of **1** was established as 4-(3,4-dihydroxyphenyl)-8-hydroxy-1,4-dihydro-3*H*-furo[3,4-*c*]chromen-3-one shown in Figure 1 and named as suillusol A. As far as we know, the novel structure represents an unprecedented type of natural product.

Compound **2**, obtained as light yellow powder, had a molecular formula of C_16_H_12_O_6_ by the negative HR-ESI-MS (*m/z* 299.0555 [M–H]^−^, calcd. 299.0561), accounting for 11 degrees of unsaturation. The ^1^H NMR spectrum (Table 2) in CD_3_OD of **2** exhibited two sets of characteristic 1,3,4-trisubstituted benzene ring signals at δ_H_ 7.38 (1H, d, *J* = 2.8 Hz, H-5), 7.37 (1H, d, *J* = 9.1 Hz, H-8), 7.16 (1H, dd, *J* = 9.1, 2.8 Hz, H-7), 6.77 (1H, d, *J* = 1.6 Hz, H-2′), 6.68 (1H, d, *J* = 8.1 Hz, H-5′), and 6.65 (1H, dd, *J* = 8.1, 1.6 Hz, H-6′), as well as a methylene singlet at δ_H_ 3.97 (2H, s, H-7′), which suggested the existence of four exchangeable protons. The ^13^C NMR spectrum (Table 2) showed a total of 16 carbon resonances, including one carbonyl carbon at δ_C_ 174.4 (s, C-4), 14 aromatic or olefinic carbons due to two benzene rings and a double bond group, as well as one aliphatic methylene carbon at δ_C_ 35.1 (t, C-7′). One carbonyl, two benzene rings, and one double bond occupied 10 degrees of unsaturation, indicative of the existence of one aliphatic ring in **2**. A detailed analysis of the HMBC correlations (Figure 2) allowed to infer the presence of a 3,4-dihydroxybenzyl moiety, which was attached to a double bond group. While the other 1,3,4-trisubstituted benzene ring was connected with the carbonyl group by the HMBC correlation from H-5 [δ_H_ 7.38 (1H, d, *J* = 2.8 Hz)] to C-4 [δ_C_ 174.4 (s)]. In addition, the ^4^*J* coupling constant of H-5 suggested a phenolic hydroxy group at C-6, which was further confirmed by analysis of the HMBC correlations (Figure 2). Finally, a comprehensive consideration of the degrees of unsaturation and the ^13^C NMR shift of C-2, C-3, and C-4 allowed us to deduce that there must be a 3-hydroxy-4*H*-pyran-4-one moiety in the structure. Therefore, the structure of **2** was established as 2-(3,4-dihydroxybenzyl)-3,6-dihydroxy-4*H*-chromen-4-one shown in Figure 1 and named as suillusol B. It is worth mentioning that compound **2**, as a representative of 2-benzylchromen-4-one, is a rare type of natural product (27).

**Figure 3:**
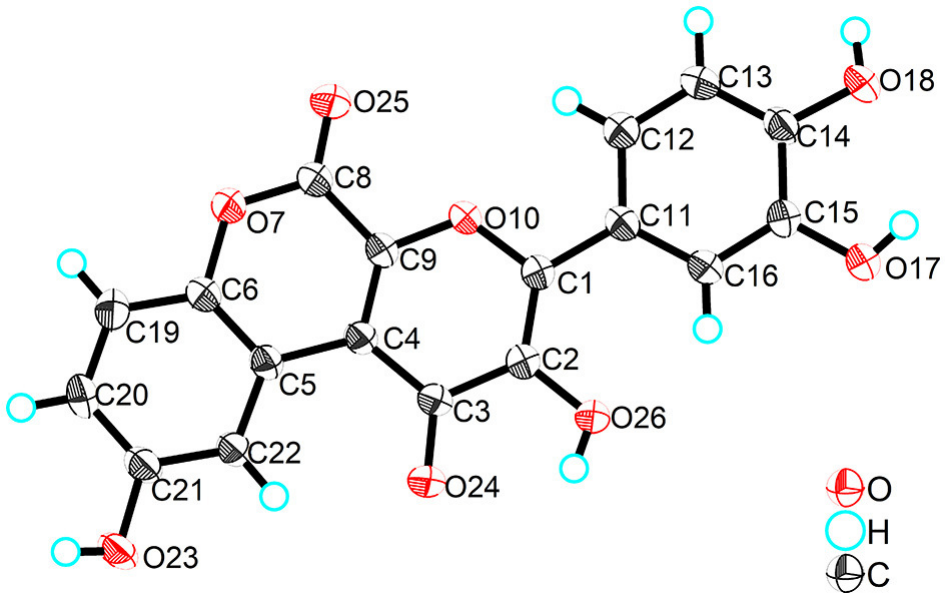
X-ray crystal structure of compound **3**.

Compound **3**, obtained as yellow crystals (methanol), had a molecular formula of C_18_H_10_O_8_ as determined by the negative HR-ESI-MS (*m/z* 353.0295 [M–H]^−^, calcd. 353.0303), accounting for 14 degrees of unsaturation. The ^1^H NMR spectrum (Table 3) in DMSO-*d*_6_ of **3** exhibited four phenolic hydroxy signals at δ_H_ 10.06 (1H, brs, 2-OH), 9.82 (1H, brs, 9-OH), 9.67 (1H, brs, 4′-OH), and 9.48 (1H, brs, 3′-OH), two sets of characteristic 1,3,4-trisubstituted benzene ring signals at δ_H_ 8.75 (1H, d, *J* = 2.9 Hz, H-10), 7.32 (1H, d, *J* = 8.9 Hz, H-7), 7.01 (1H, dd, *J* = 8.9, 2.9 Hz, H-8), 7.72 (1H, d, *J* = 2.2 Hz, H-2′), 7.61 (1H, dd, *J* = 8.5, 2.2 Hz, H-6′), and 6.93 (1H, d, *J* = 8.5 Hz, H-5′). The ^13^C NMR spectrum (Table 3) showed a total of 18 carbon resonances, and all were in the range of 111.6–172.0 ppm, including six aromatic methine carbons and 12 quaternary ones. The aforementioned NMR features suggested that **3** should be a polyphenol containing two benzene rings. A detailed analysis of the HMBC correlations (Figure 2) allowed to establish the specific substitutions of two benzene ring moieties. However, the four carbon signals at δ_C_ 172.0, 154.7, 142.6, and 142.5 were still unsolved, since no correlations were observed in the HMBC spectrum. Thereupon we had to resort to using single crystal X-ray diffraction method. Fortunately, its single crystals were eventually obtained through multiple attempts, and the structure was conclusively determined to be 3-(3,4-dihydroxyphenyl)-2,9-dihydroxypyrano[2,3-*c*]chromene-1,5-dione by single crystal X-ray crystallographic analysis (Figure 3). Thus, the structure of **3** was established as shown in Figure 1 and named as suillusol C. As far as we know, compound **3** represents a new type of polyphenol fungal pigment, which may be biosynthetically related to thelephoric acid.

Compound **4**, isolated as a yellow powder, possessed a molecular formula of C_17_H_10_O_6_ by the positive HR-ESI-MS (*m/z* 311.0542 [M+H]^+^, calcd. 311.0550), indicating 13 degrees of unsaturation. The ^1^H NMR spectrum (Table 4) in DMSO-*d*_6_ of **4** showed a group of *p*-substituted benzene ring signals at δ_H_ 7.92 (2H, d, *J* = 8.7 Hz, H-2′/6′) and 6.89 (2H, d, *J* = 8.7 Hz, H-3′/5′), as well as three aromatic or olefinic proton singlets at δ_H_ 7.22 (1H, s, H-9), 7.03 (1H, s, H-5), and 7.00 (1H, s, H-8), which hinted the existence of three phenolic hydroxy protons. The ^13^C NMR spectrum (Table 4) showed a total of 17 carbon resonances, including a conjugated lactone carbonyl carbon at δ_C_ 166.6 (s, C-2), as well as 16 aromatic or olefinic carbons assignable to two benzene rings and two double bond groups. The analysis of the degrees of unsaturation hinted the existence of two additional rings. An analysis of the HMBC correlations (Figure 2) revealed the presence of a 4-hydroxyphenyl moiety, which was located at an up-field *sp* carbon at δ_C_ 94.3 (s, C-3). According to the peak shape of the remaining proton signals, the other benzene ring was inferred as 1,2,4,5-tetrasubstituted and located on a chromene nucleus, which was supported by the observable HMBC correlations from H-5 [δ_H_ 7.03 (1H, s)] to C-7 [δ_C_ 143.7 (s)] and C-8a [δ_C_ 111.7 (s)], from H-8 [δ_H_ 7.00 (1H, s)] to C-4a [δ_C_ 144.3 (s)], C-6 [δ_C_ 148.1 (s)] and C-9 [δ_C_ 106.4 (d)], and from H-9 [δ_H_ 7.22 (1H, s)] to C-3a [δ_C_ 155.5 (s)], C-4a and C-8 [δ_C_ 112.8 (d)]. By consideration of the ^13^C NMR shifts of the unsolved carbons and the degrees of unsaturation, the other ring system was assigned to a conjugated γ-lactone unit, which was further fused by the chromene nucleus to form an unusual 2*H*-furo[3,2-*b*]chromen-2-one core. And then two phenolic hydroxy groups were necessarily attached at C-6 and C-7. Therefore, the structure of **4** was established as 6,7-dihydroxy-3-(4-hydroxyphenyl)-2*H*-furo[3,2-*b*]chromen-2-one, shown in Figure 1 and named as suillusol D.

Compound **5**, isolated as light yellow powder, possessed a molecular formula of C_18_H_12_O_8_ by the negative HR-ESI-MS (*m/z* 355.0458 [M–H]^−^, calcd. 355.0459), indicating 13 degrees of unsaturation. The ^1^H NMR spectrum (Table 5) in CD_3_OD of **5** exhibited two groups of 1,3,4-trisubstituted benzene ring signals at δ_H_ 7.77 (1H, brs, H-2′), 7.62 (1H, brd, *J* = 8.4 Hz, H-6′), 6.77 (1H, d, *J* = 8.4 Hz, H-5′), 6.79 (1H, d, *J* = 2.2 Hz, H-8), 6.65 (1H, d, *J* = 8.6 Hz, H-5), and 6.58 (1H, dd, *J* = 8.6, 2.2 Hz, H-6), as well as one downfield aliphatic methine singlet at δ_H_ 3.94 (1H, brs, H-8b), which hinted the existence of five exchangeable protons. The ^13^C NMR spectrum (Table 5) showed a total of 18 carbon resonances, including one conjugated ketone carbon at δ_C_ 200.2 (s, C-1), one carboxylic carbon at δ_C_ 178.1 (s, C-9), 14 aromatic or olefinic carbons assignable to two benzene rings and a double bond group, one downfield oxygenated *sp*^3^ quaternary carbon at δ_C_ 93.7 (s, C-3a), as well as one aliphatic methine carbon at δ_C_ 57.7 (d, C-8b). The analysis of the degrees of unsaturation was indicative of the presence of two aliphatic rings in **5**. The aforementioned NMR features were very similar to those of suillusin, a unique cyclopenta[*b*]benzofuran derivative isolated from the same genus (19). Comparison of the NMR data of **5** with those of suillusin revealed that the signal differences were mainly from the carboxylic acid group at C-3a. The absence of the methoxy signals and the obvious downfield shift of the carboxylic carbon indicated that the methoxycarbonyl group was replaced by a carboxylic one in **5**. The resulting structure was further verified by the careful HMBC analysis (Figure 2). While the relative configuration of two chiral centers was deduced to be the same as that of suillusin by comparison of their NMR data. Therefore, the structure of **5** was established as shown in Figure 1 and named as suillusinoic acid.

Compound **6**, light yellow powder, possessed a molecular formula of C_20_H_16_O_8_ by the negative HR-ESI-MS (*m/z* 383.0762 [M–H]^−^, calcd. 383.0772). The ^1^H and ^13^C NMR spectra (Table 5) in CD_3_OD of **6** were very similar to those of suillusinoic acid (5). Comparison of the NMR data of **6** with those of **5** revealed that the signal differences were also from the carboxylic acid moiety at C-3a. The appearance of the ethoxy signals and the obvious upfield shift of the carboxylic carbon indicated that the carboxylic group was ethylated in **6**, which was confirmed by the observable HMBC correlations from H_2_-1′' [δ_H_ 4.16–4.22 (2H, m)] and H-8b [δ_H_ 4.12 (1H, brs)] to the ester carbonyl carbon [δ_C_ 172.9 (s, C-9)]. Similarly, the relative configuration was the same as that of suillusinoic acid by comparison of their NMR data. Thus, the structure of **6** was established as shown in Figure 1 and named as ethyl suillusinoate.

### Cytotoxicity against HepG2 cells

**Figure 4:**
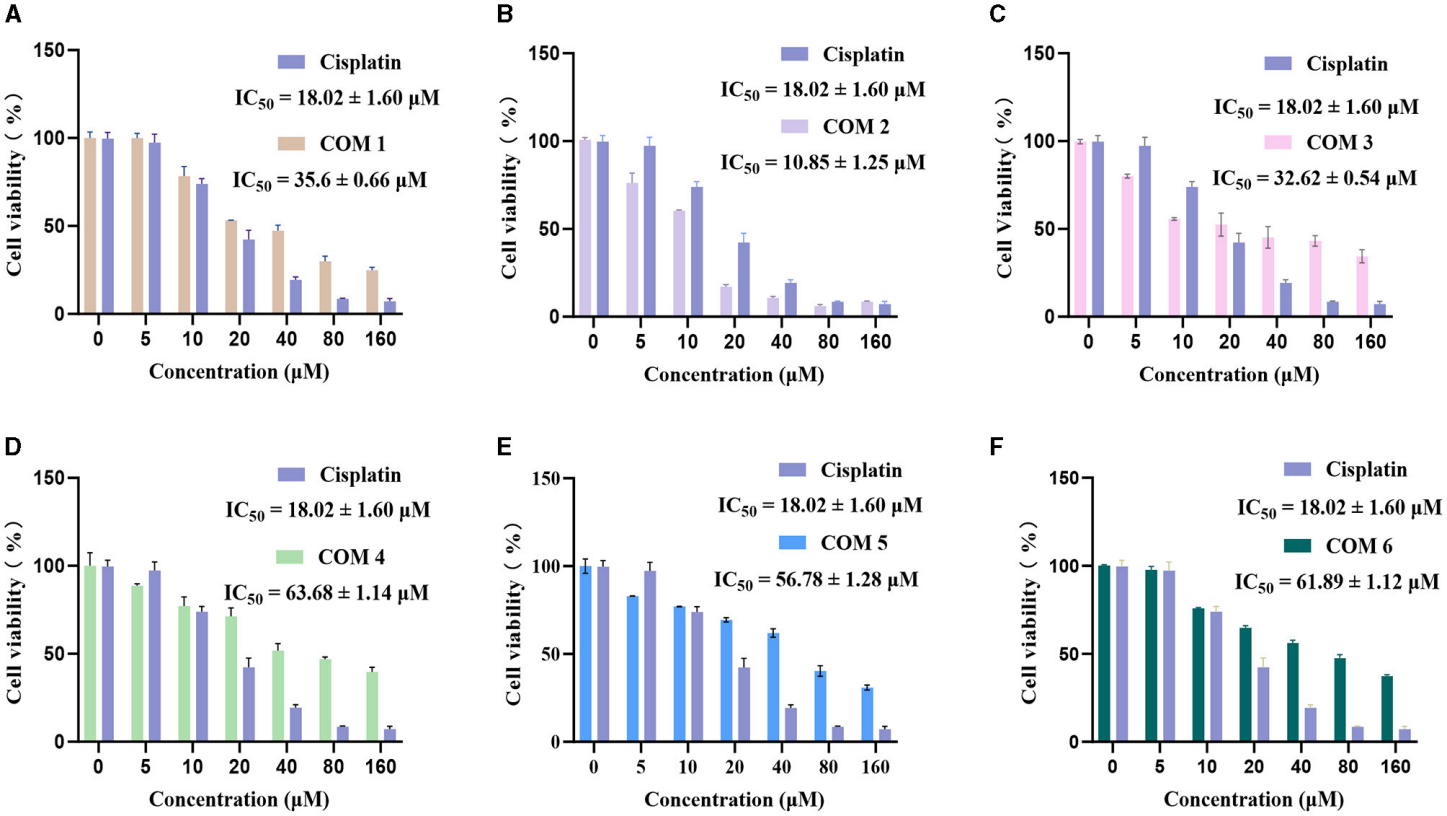
Inhibitory effects of compounds **1**–**6** on the HepG2 (**A**–**F**). Compounds **1**–**6**.

We tested the antitumor activity against HepG2 human liver carcinoma cells of the isolated compounds **1**–**6**. The results shown in Figure 4 indicated that compound **2** exhibit higher efficacy than cisplatin, a commonly used anticancer chemotherapeutic agent, and demonstrate exceptional antitumor activity, with the half-maximal inhibitory concentrations (IC_50_) measured at 10.85 μM. Additionally, compounds **1** and **3** also exhibit moderate antitumor activity, with IC_50_ values of 35.60 and 32.62 μM. Compounds **5** and **6** exhibited the lowest inhibitory activity, with their IC_50_ values showing no significant difference due to their structural similarity. But during the experimental process, compounds **5** and **6** displayed color-changing phenomena, suggesting that they may have a more pronounced effect in antioxidant activity.

## Conclusion

Six new polyphenolic compounds, named suillusol A-D (**1**–**4**), suillusinoic acid (**5**), ethyl suillusinoate (**6**), were isolated from the macrofungus *S. granulatus*. All the isolated compounds exhibited some degree of cytotoxicity against HepG2 cells. Compound **2** showed improved inhibitory activities than positive control cisplatin. Moreover, compounds **1** and **3** also exhibited moderate cytotoxic activity against HepG2 cells. Those results suggested that the polyphenolic compounds isolated from the *S. granulatus* could be investigated as natural anticancer agent in the pharmaceutical and food industries.

## Data availability statement

The original contributions presented in the study are included in the article/Supplementary material, further inquiries can be directed to the corresponding authors.

## Author contributions

HZ: Conceptualization, Project administration, Visualization, Writing – review & editing. MX: Resources, Validation, Visualization, Writing – review & editing. XY: Conceptualization, Formal analysis, Methodology, Software, Writing – review & editing. LY: Investigation, Methodology, Resources, Validation, Writing – original draft. ZW: Investigation, Writing – review & editing. L-aW: Formal analysis, Methodology, Resources, Software, Validation, Writing – original draft. ZL: Conceptualization, Formal analysis, Supervision, Visualization, Writing – review & editing. JZ: Data curation, Funding acquisition, Investigation, Methodology, Project administration, Writing – original draft. JL: Conceptualization, Methodology, Writing – original draft, Writing – review & editing.
