# Supplementary material for: Six new polyphenolic metabolites isolated from the Suillus granulatus and their cytotoxicity against HepG2 cells
Source: Front Nutr. 2024 Apr 24;11:1390256. doi: 10.3389/fnut.2024.1390256 (PMC11076869; doi:10.3389/fnut.2024.1390256)
Supplement: Supplementary file 1 [file Image_1.pdf]

Supplementary Material

Six New Polyphenolic Metabolites Isolated from the *Suillus granulatus* and Their Cytotoxicity against HepG2 Cells

Hanyu Zhao[1], Miaomiao Xiong[1], Xiaomin Yang[1], Lan Yao[2], Li-an Wang[1,3], Zhuang Li[1], Jinxiu Zhang[1*], Jianhua Lv[1*]

[1]College of Life Sciences, Hebei Normal University, Shijiazhuang, China

[2]Institute of Biology, Hebei Academy of Science, Shijiazhuang, China

[3]Hebei Collaborative Innovation Center for Eco-Environment, Shijiazhuang, China

***Correspondence:**

Jianhua Lv

lvjianhua@hebtu.edu.cn

Jinxiu Zhang

xiudou882003@163.com

List of Supplementary Material

Figure S1. ^{1}H-NMR spectrum of compound **1**

Figure S2. ^{13}C-NMR spectrum of compound **1**

Figure S3. HSQC spectrum of compound **1**

Figure S4. HMBC spectrum of compound **1**

Figure S5. ^{1}H-^{1}H COSY spectrum of compound **1**

Figure S6. ^{1}H-NMR spectrum of compound **2**

Figure S7. ^{13}C-NMR spectrum of compound **2**

Figure S8. HSQC spectrum of compound **2**

Figure S9. HMBC spectrum of compound **2**

Figure S10. ^{1}H-^{1}H COSY spectrum of compound **2**

Figure S11. ^{1}H-NMR spectrum of compound **3**

Figure S12. ^{13}C-NMR spectrum of compound **3**

Figure S13. HSQC spectrum of compound **3**

Figure S14. HMBC spectrum of compound **3**

Figure S15. ^{1}H-^{1}H COSY spectrum of compound **3**

Figure S16. ^{1}H-NMR spectrum of compound **4**

Figure S17. ^{13}C-NMR spectrum of compound **4**

Figure S18. HSQC spectrum of compound **4**

Figure S19. HMBC spectrum of compound **4**

Figure S20. ^{1}H-^{1}H COSY spectrum of compound **4**

Figure S21. ^{1}H-NMR spectrum of compound **5**

Figure S22. ^{13}C-NMR spectrum of compound **5**

Figure S23. HSQC spectrum of compound **5**

Figure S24. HMBC spectrum of compound **5**

Figure S25. ^{1}H-^{1}H COSY spectrum of compound **5**

Figure S26. NOESY spectrum of compound **5**

Figure S27. ^{1}H-NMR spectrum of compound **6**

Figure S28. ^{13}C-NMR spectrum of compound **6**

Figure S29. HSQC spectrum of compound **6**

Figure S30. HMBC spectrum of compound **6**

Figure S31. ^{1}H-^{1}H COSY spectrum of compound **6**

Figure S32. NOESY spectrum of compound **6**

Figure S33. HR-ESI-MS of compound **1**

Figure S34. HR-ESI-MS of compound **2**

Figure S35. HR-ESI-MS of compound **3**

Figure S36. HR-ESI-MS of compound **4**

Figure S37. HR-ESI-MS of compound **5**

Figure S38. HR-ESI-MS of compound **6**

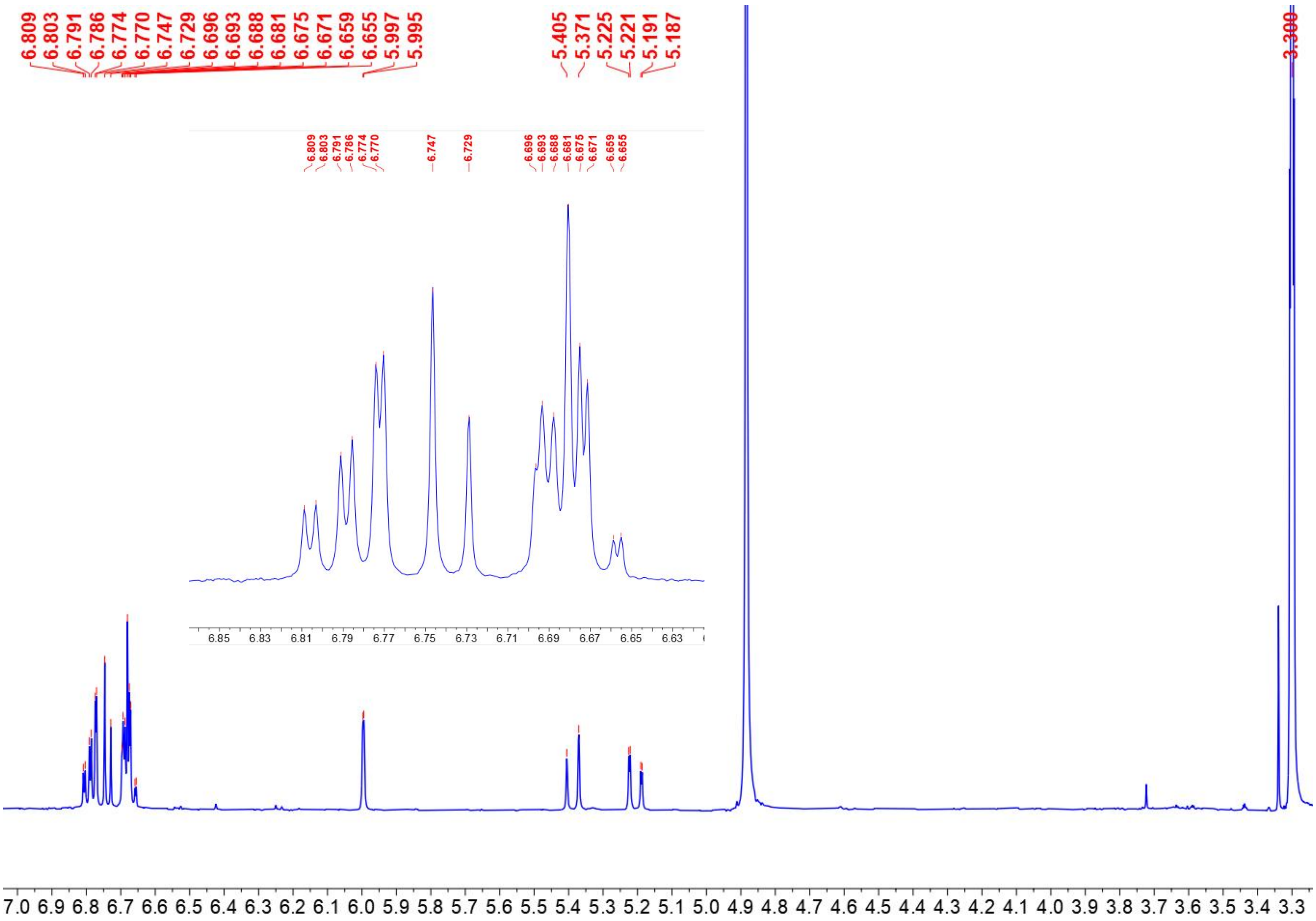

Figure S1. ^{1}H-NMR spectrum of compound **1**

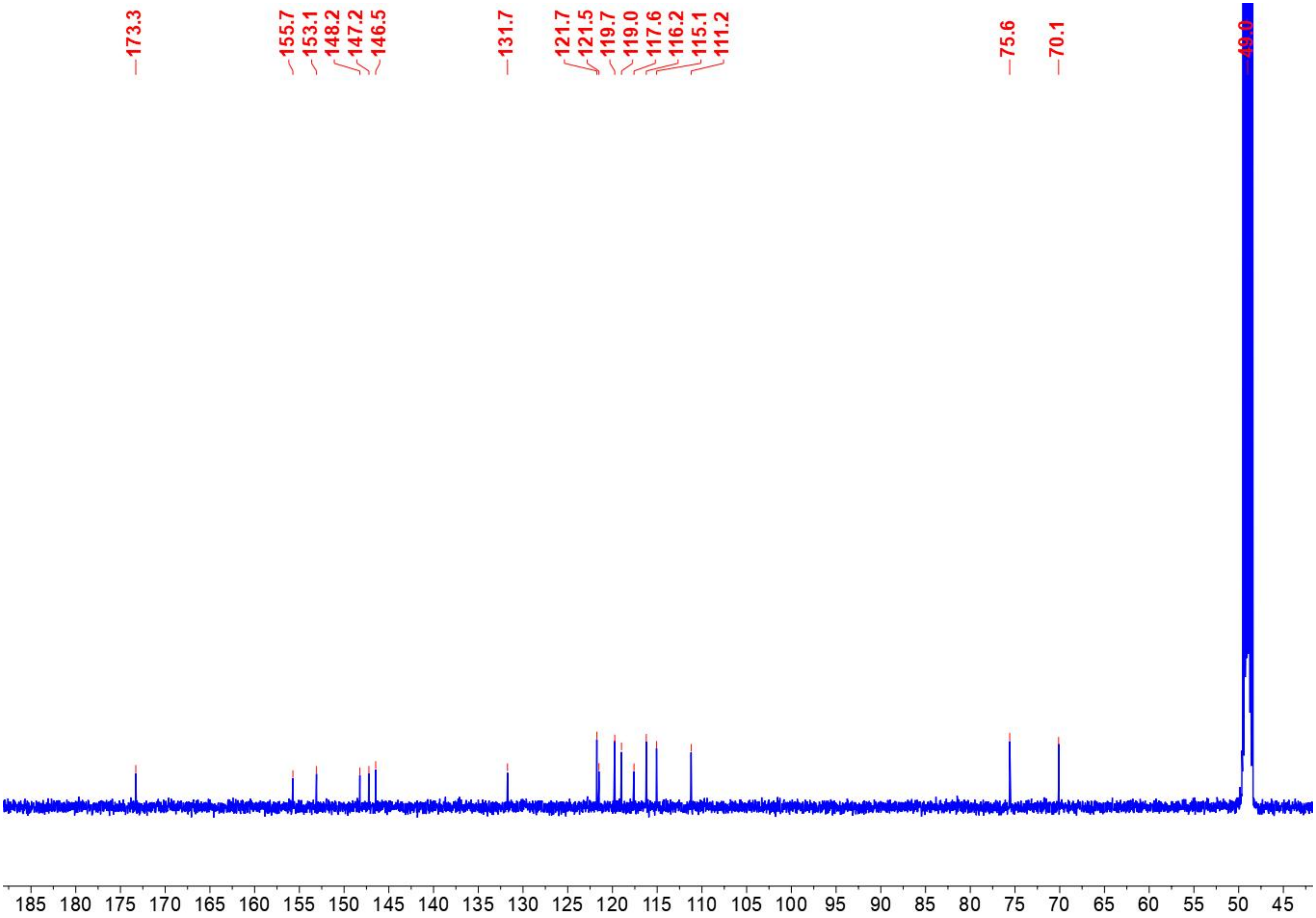

Figure S2. ^{13}C-NMR spectrum of compound **1**

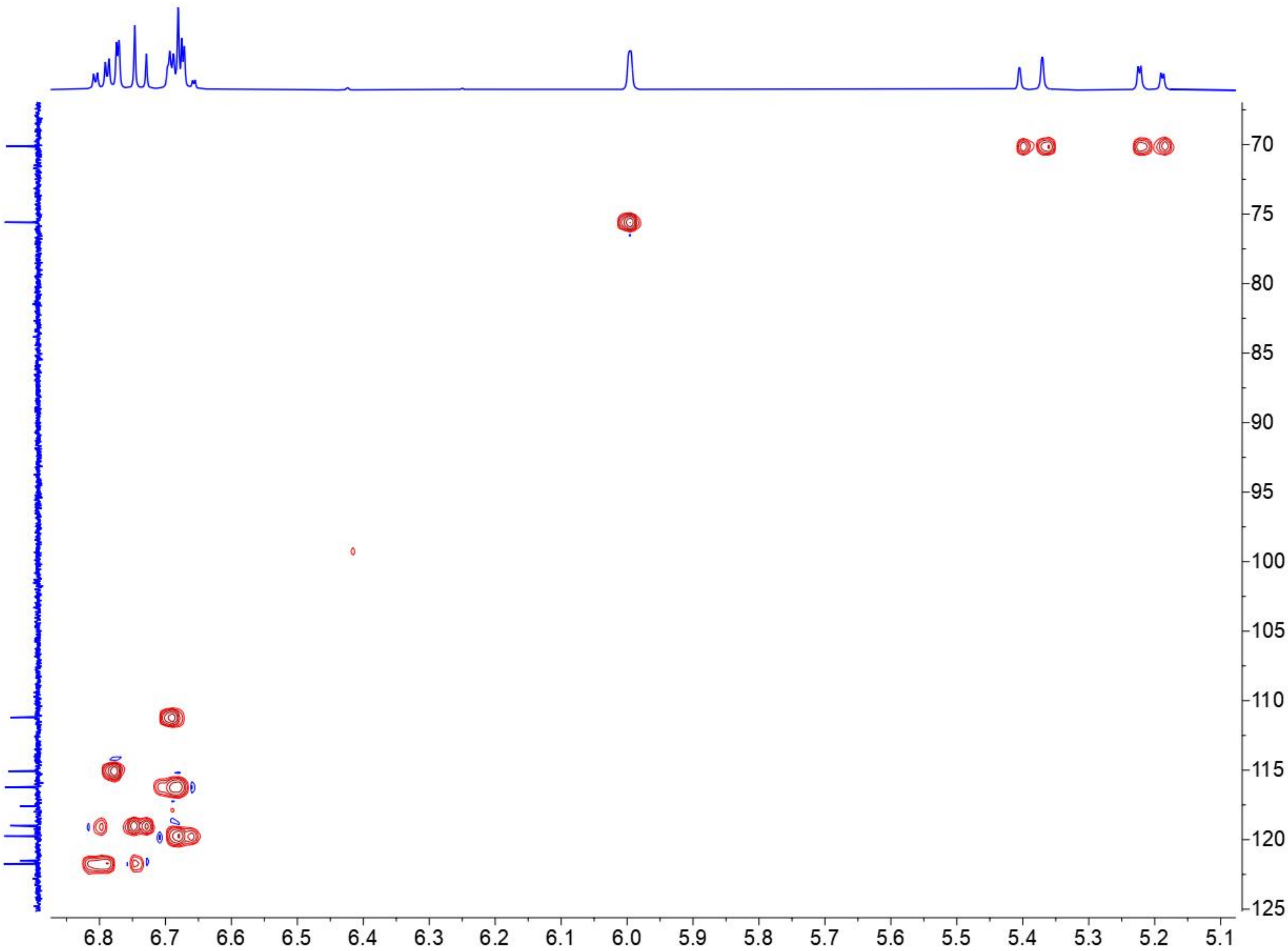

Figure S3. HSQC spectrum of compound **1**

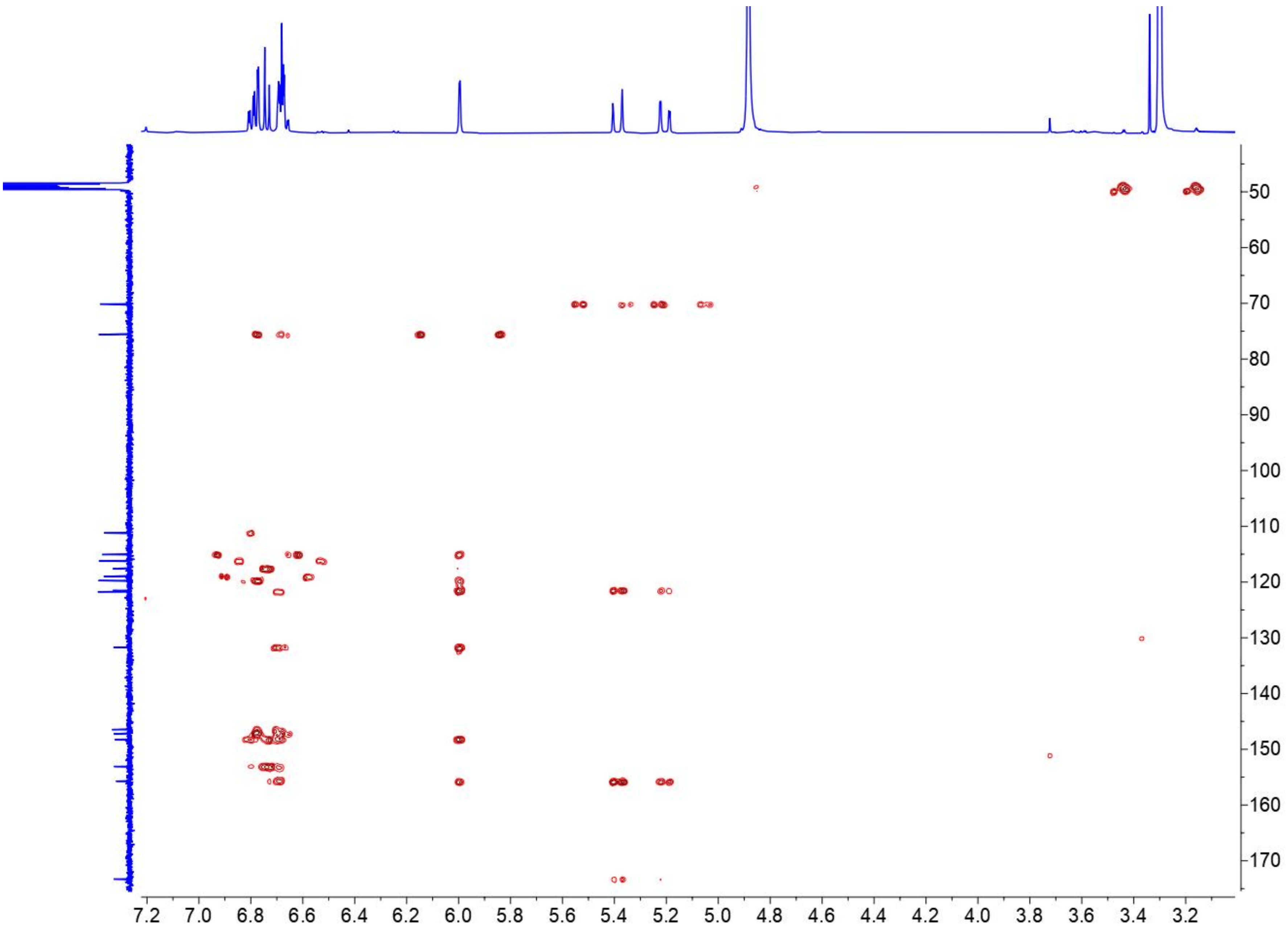

Figure S4. HMBC spectrum of compound **1**

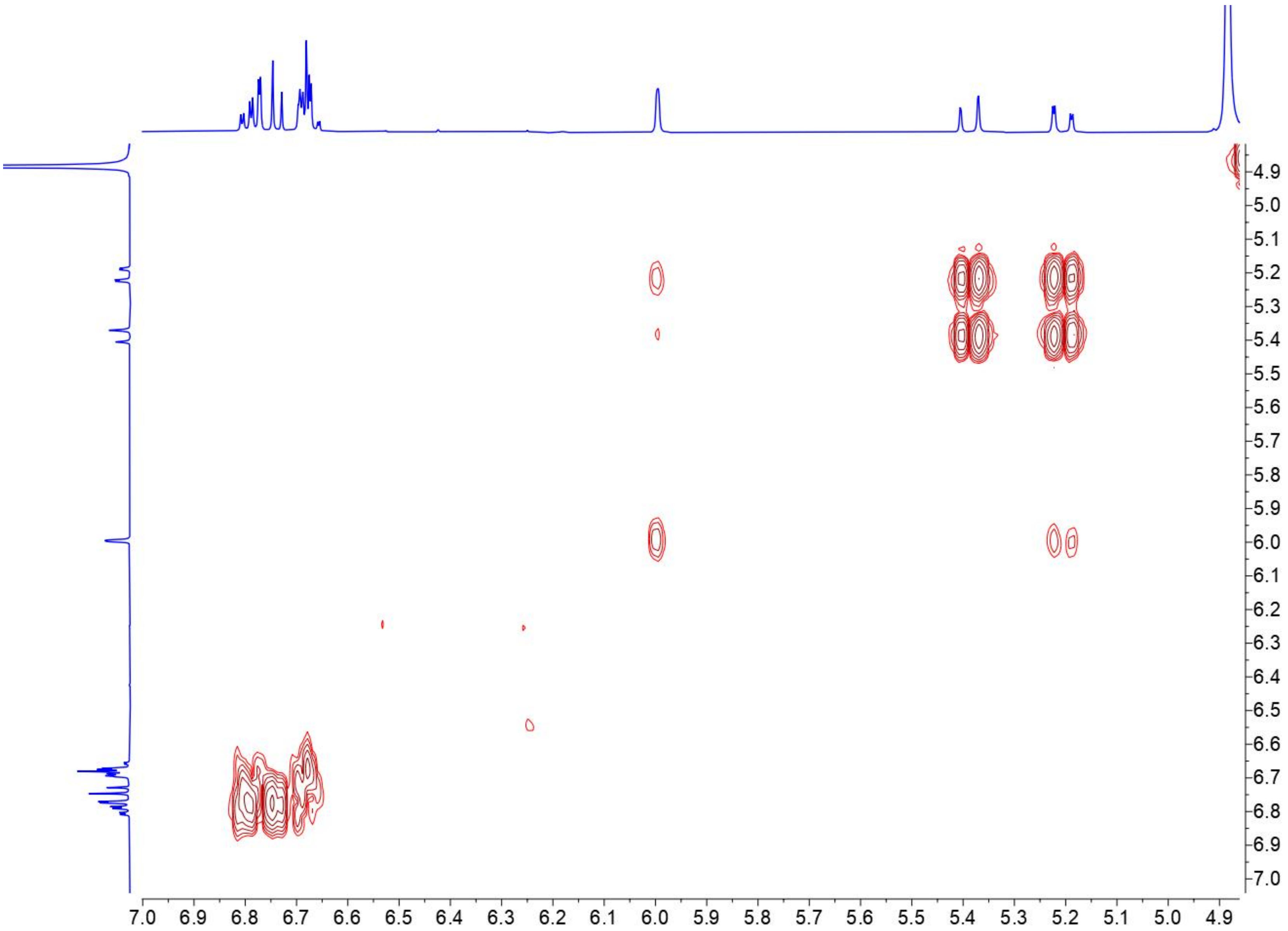

Figure S5. ^{1}H-^{1}H COSY spectrum of compound **1**

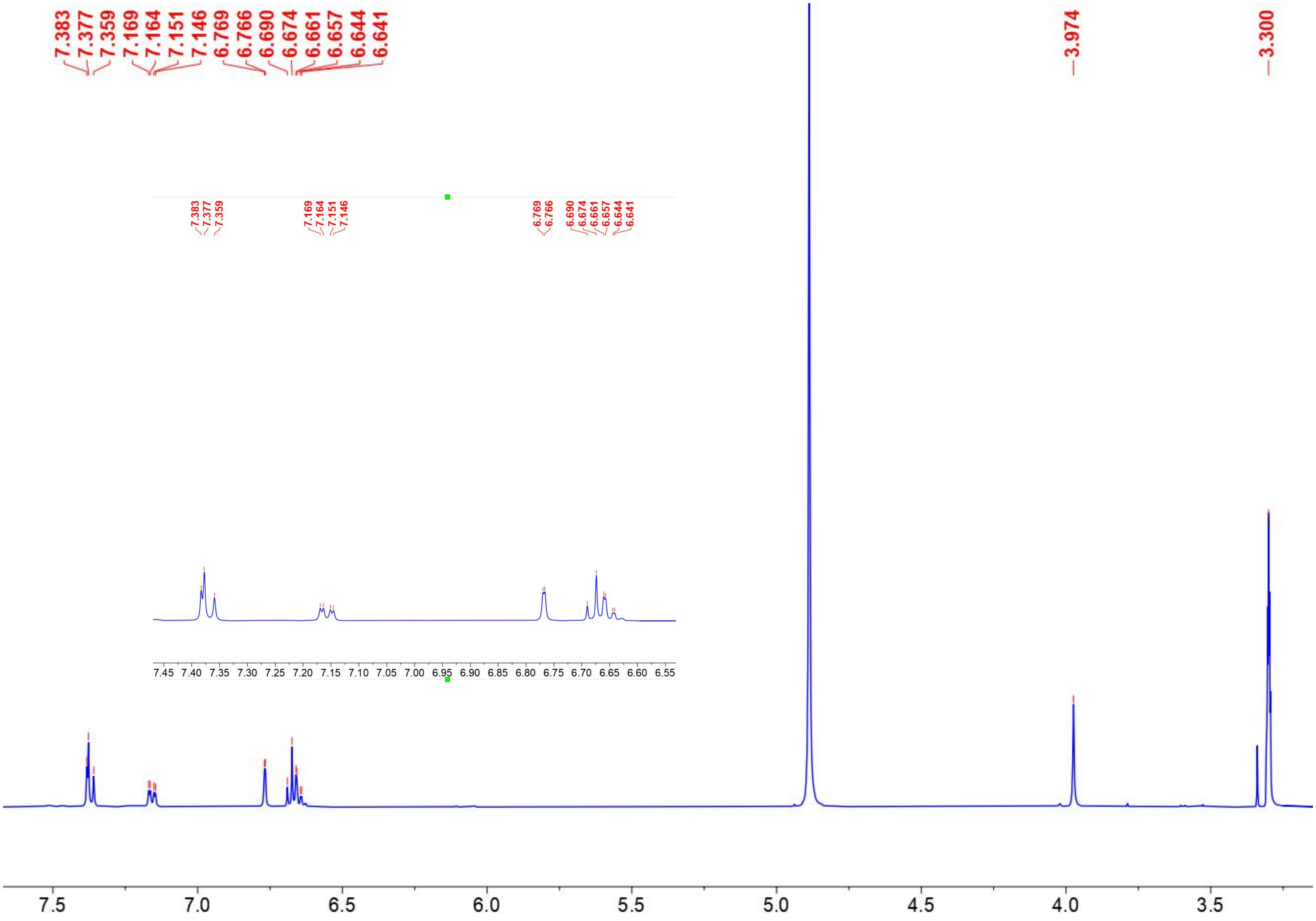

Figure S6. ^{1}H-NMR spectrum of compound **2**

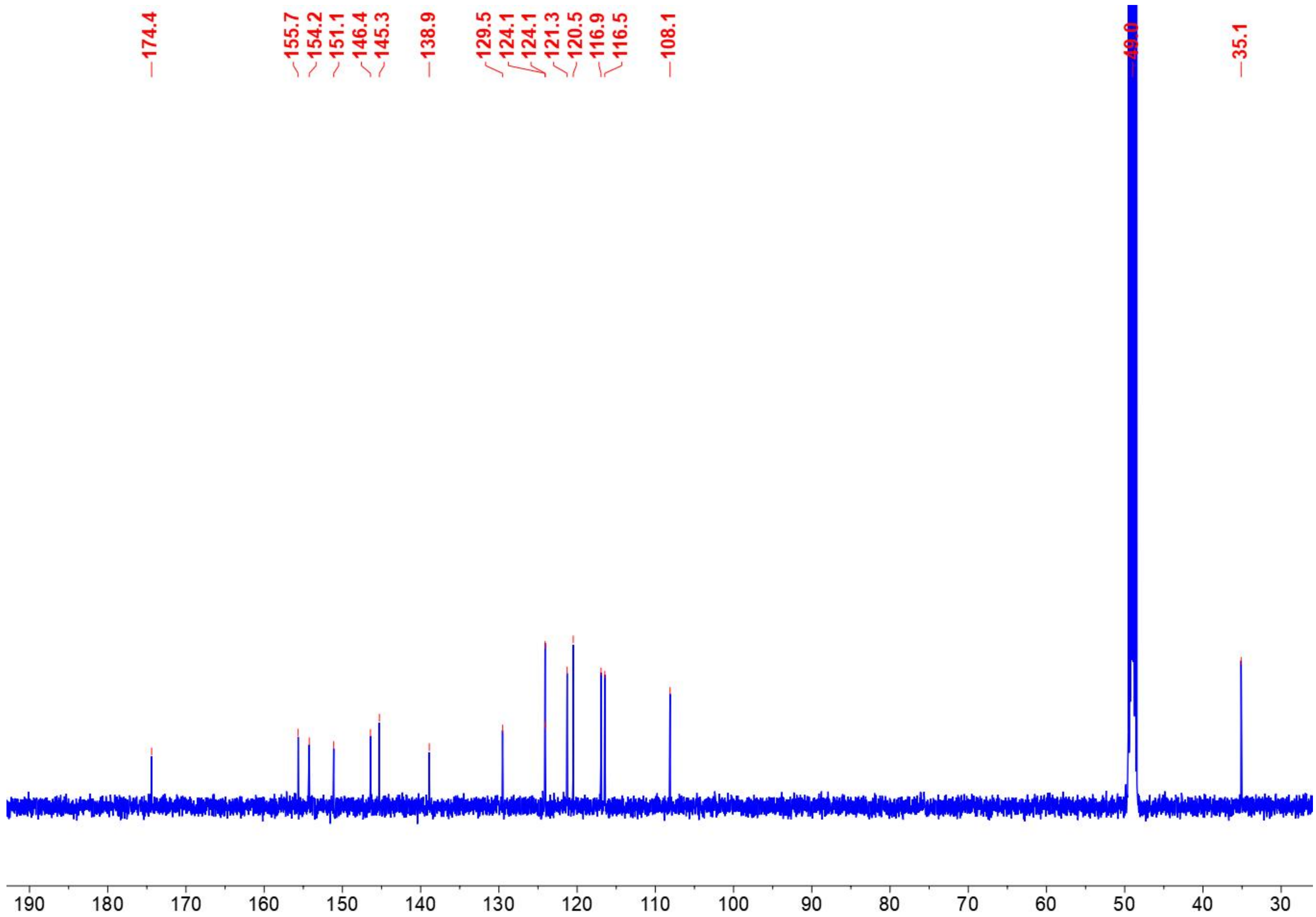

Figure S7. ^{13}C-NMR spectrum of compound **2**

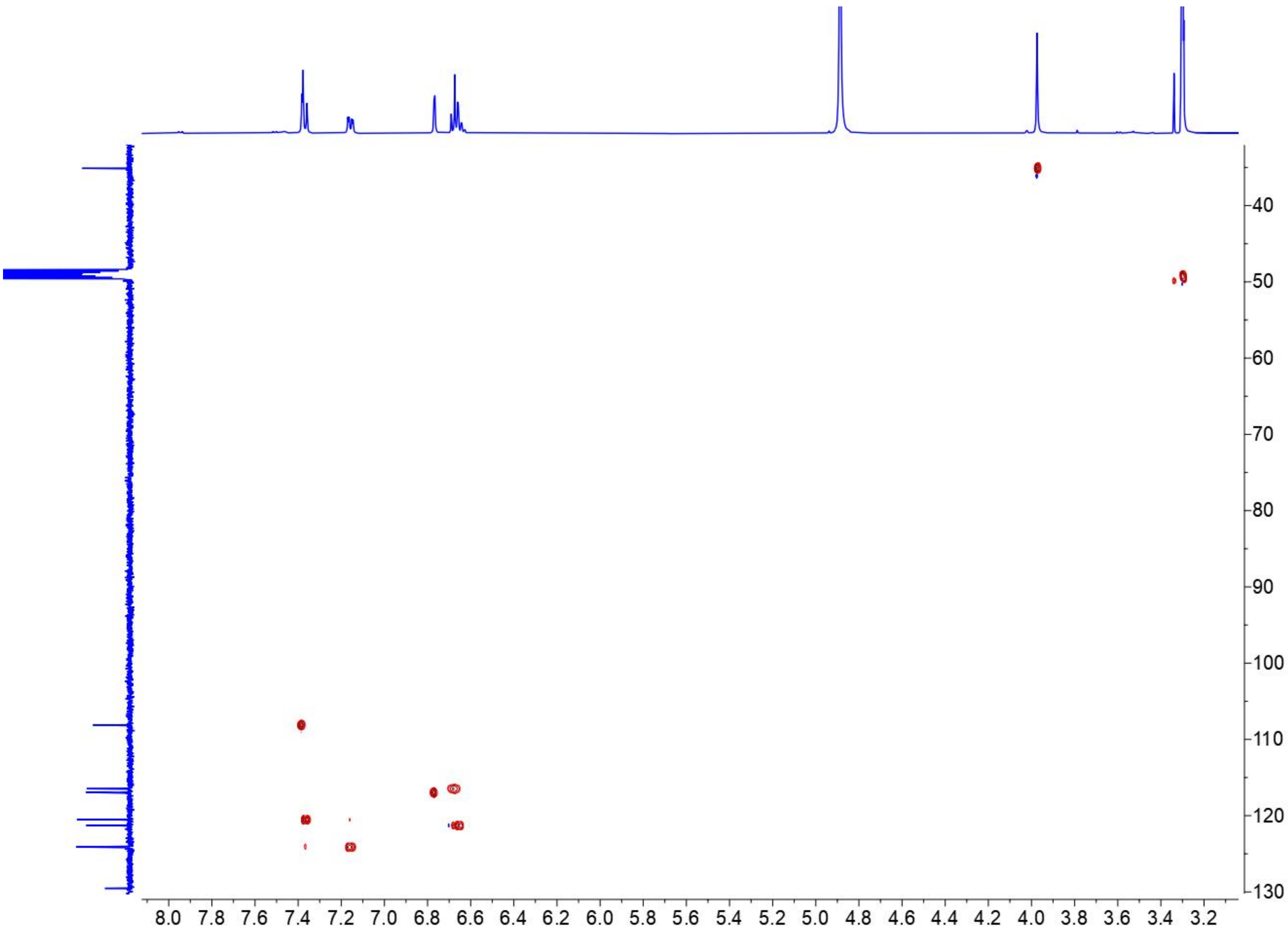

Figure S8. HSQC spectrum of compound **2**

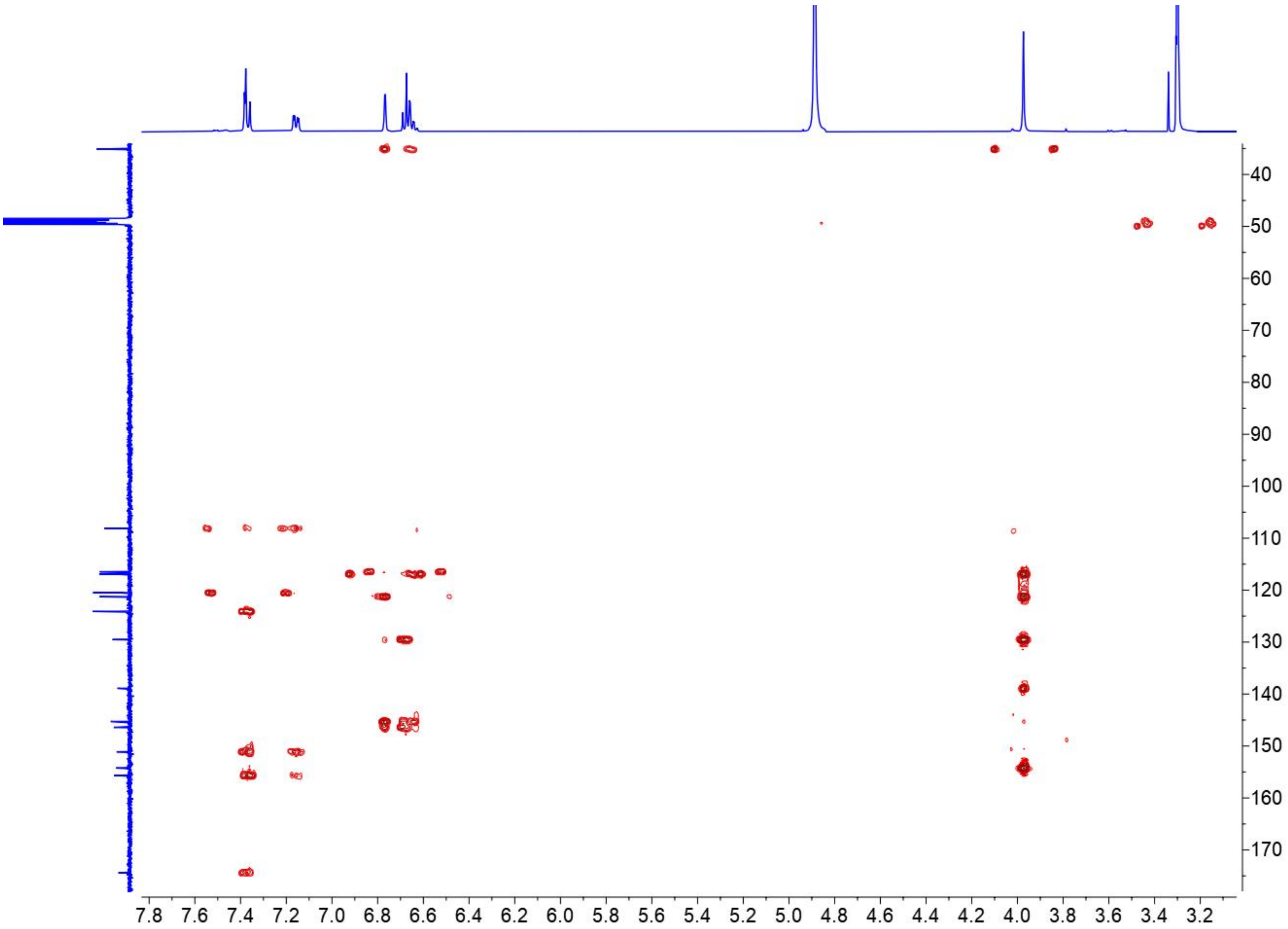

Figure S9. HMBC spectrum of compound **2**

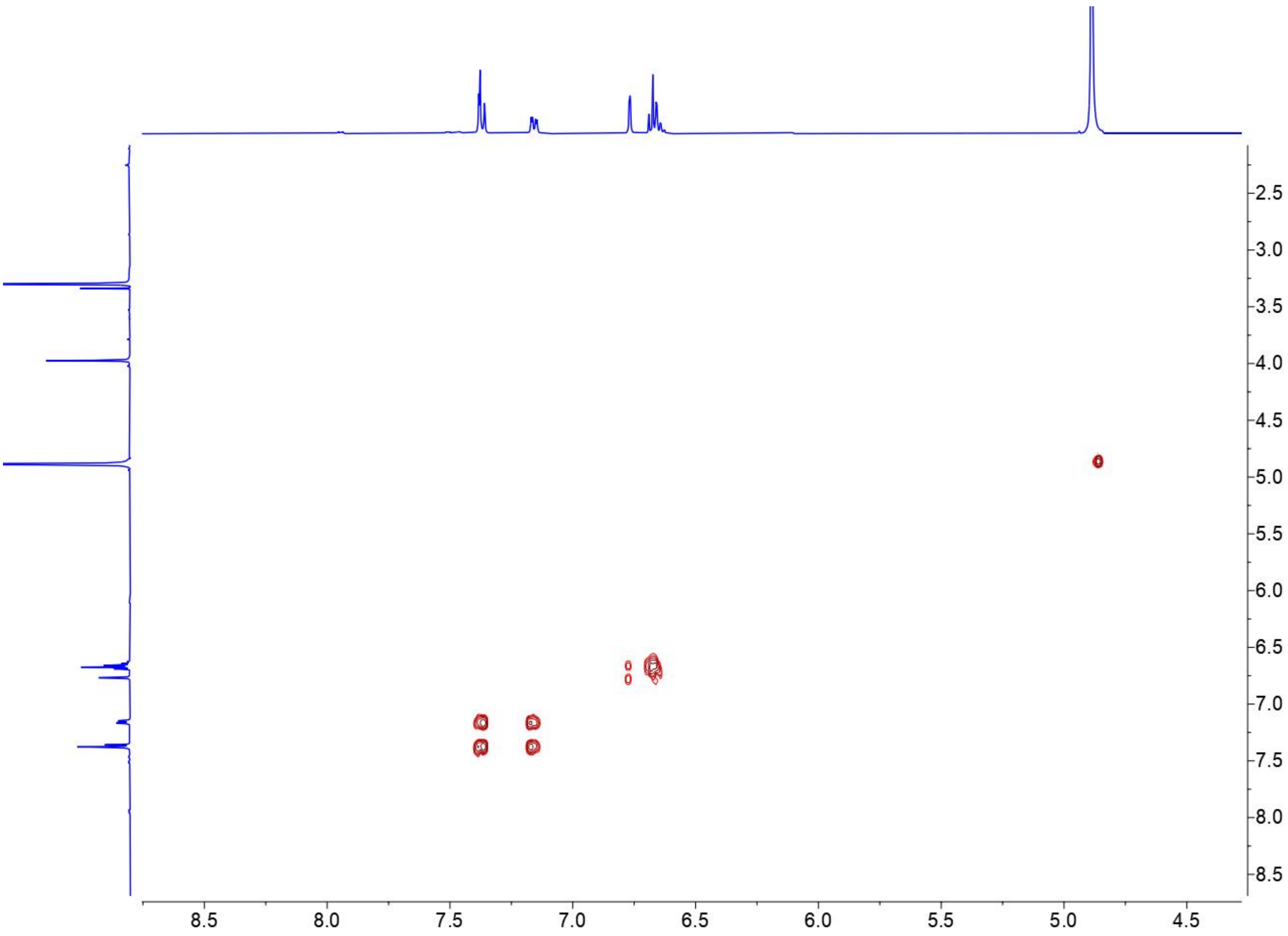

Figure S10. ^{1}H-^{1}H COSY spectrum of compound **2**

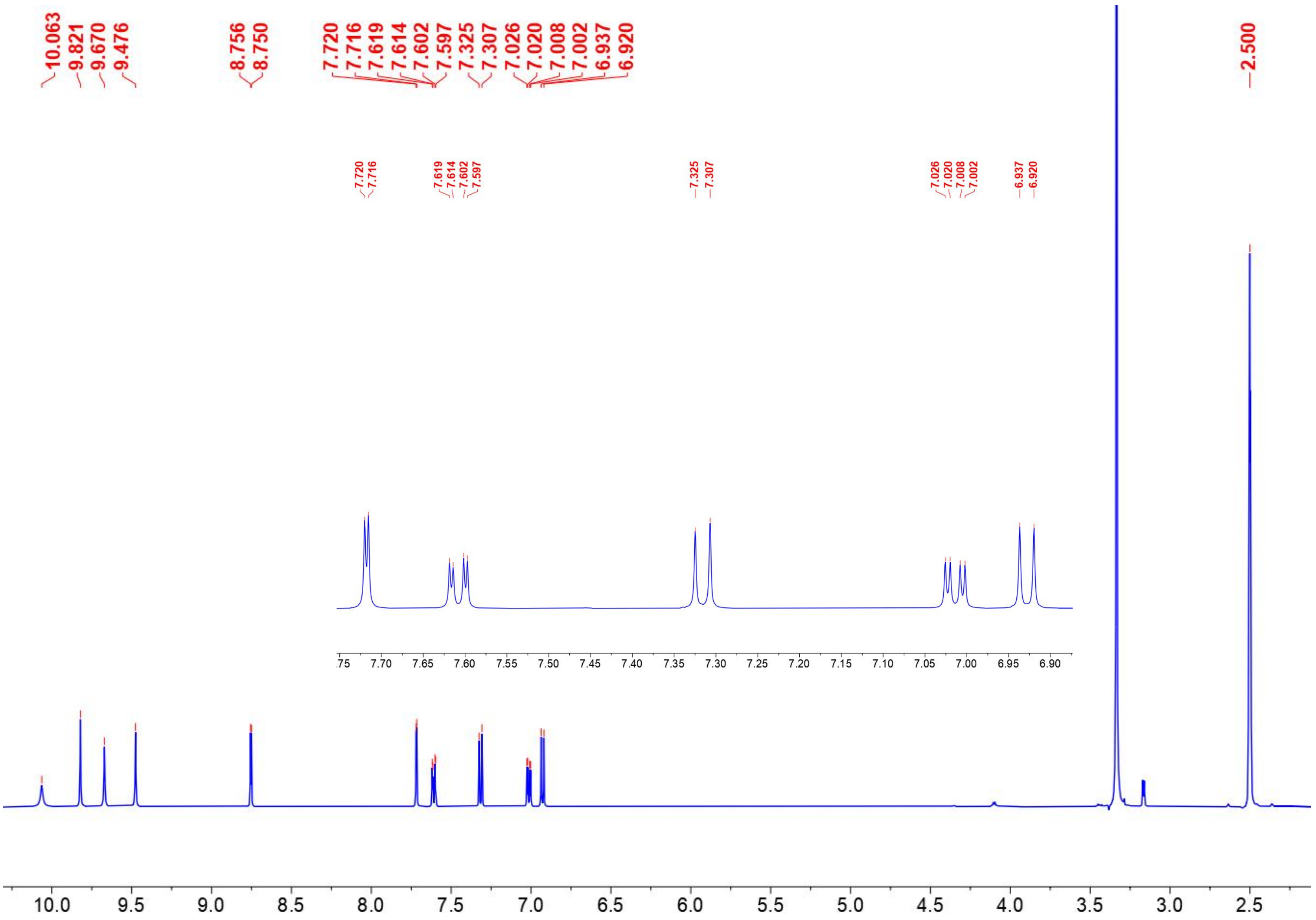

Figure S11. ^{1}H-NMR spectrum of compound **3**

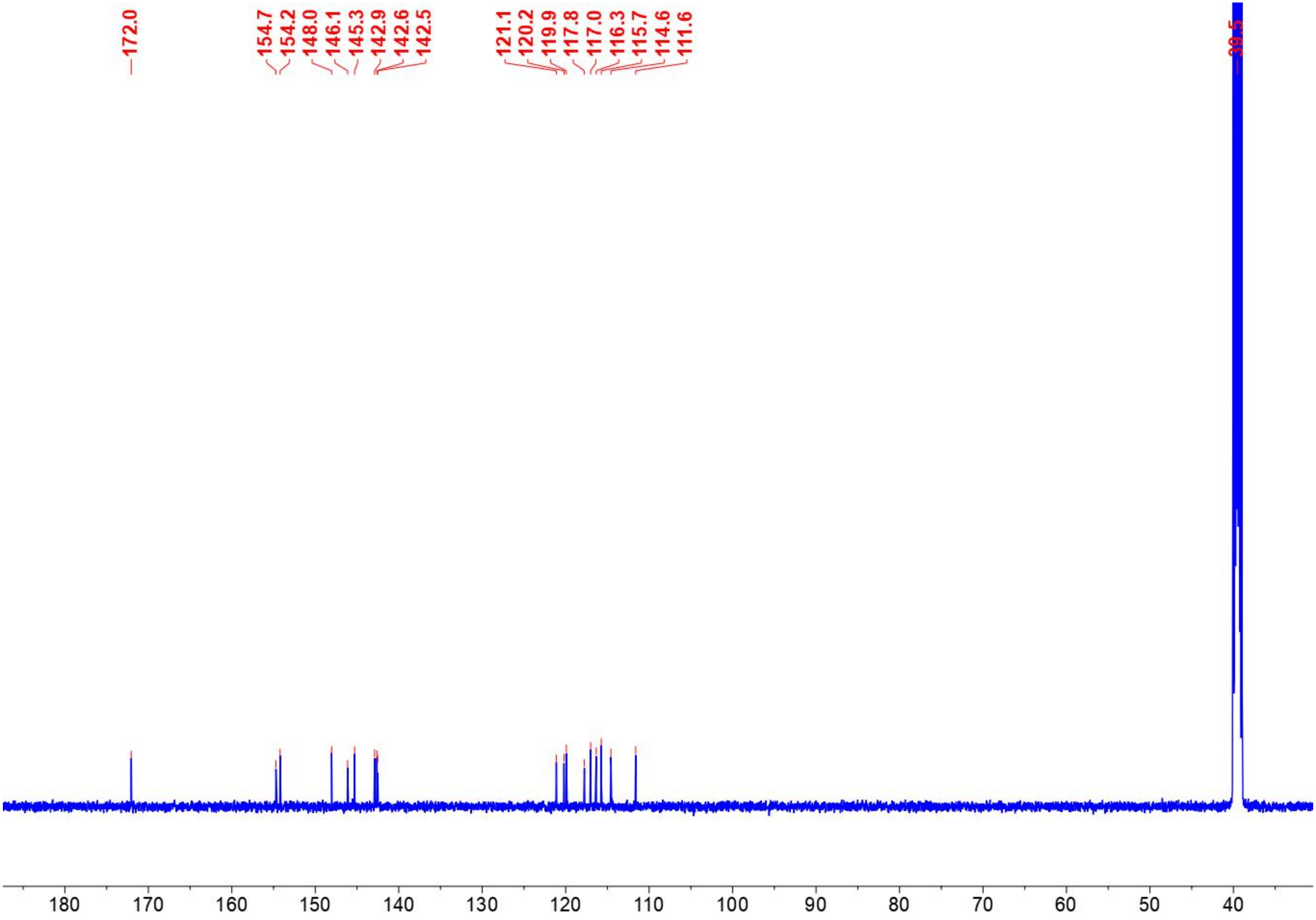

Figure S12. ^{13}C-NMR spectrum of compound **3**

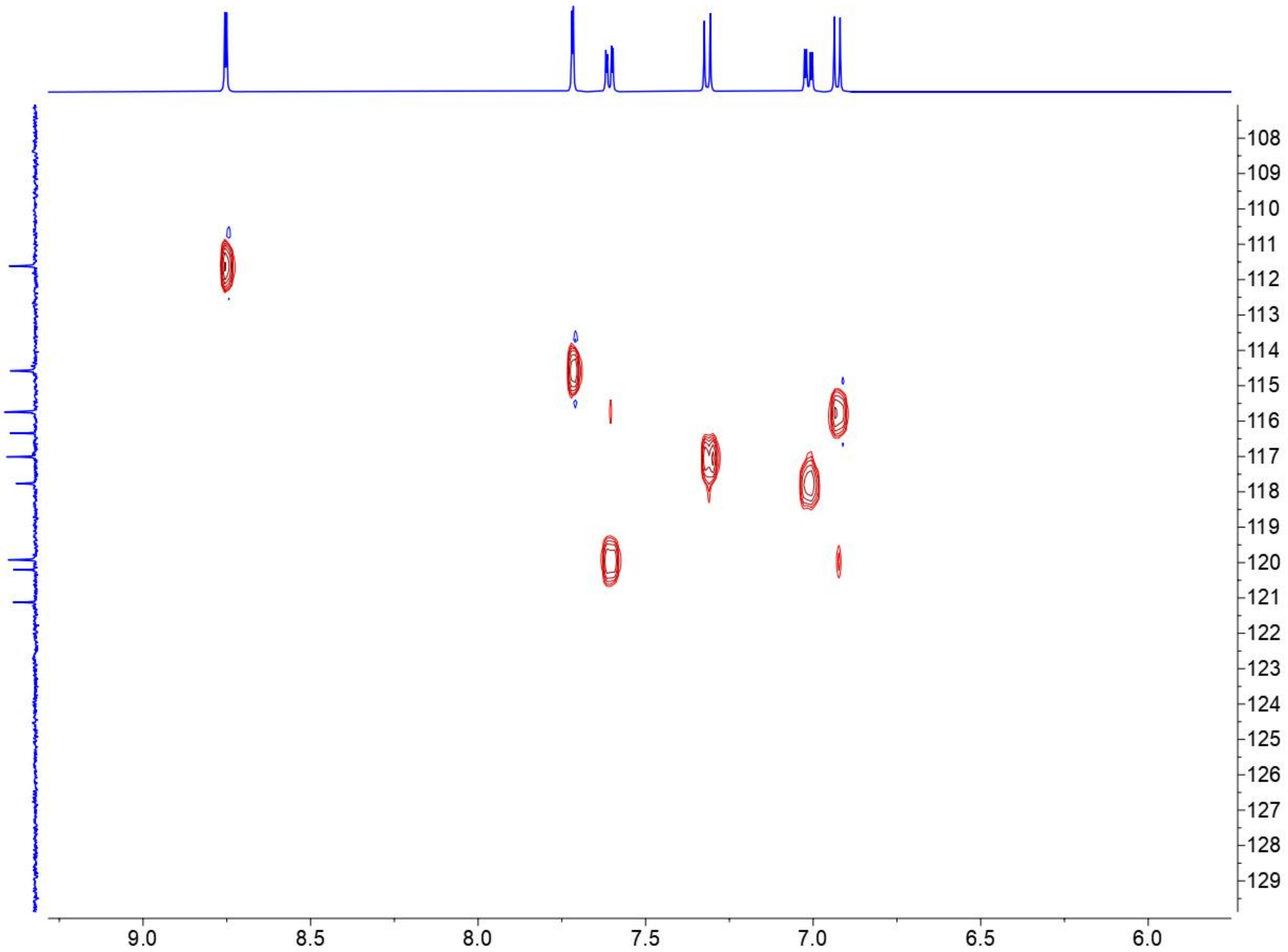

Figure S13. HSQC spectrum of compound **3**

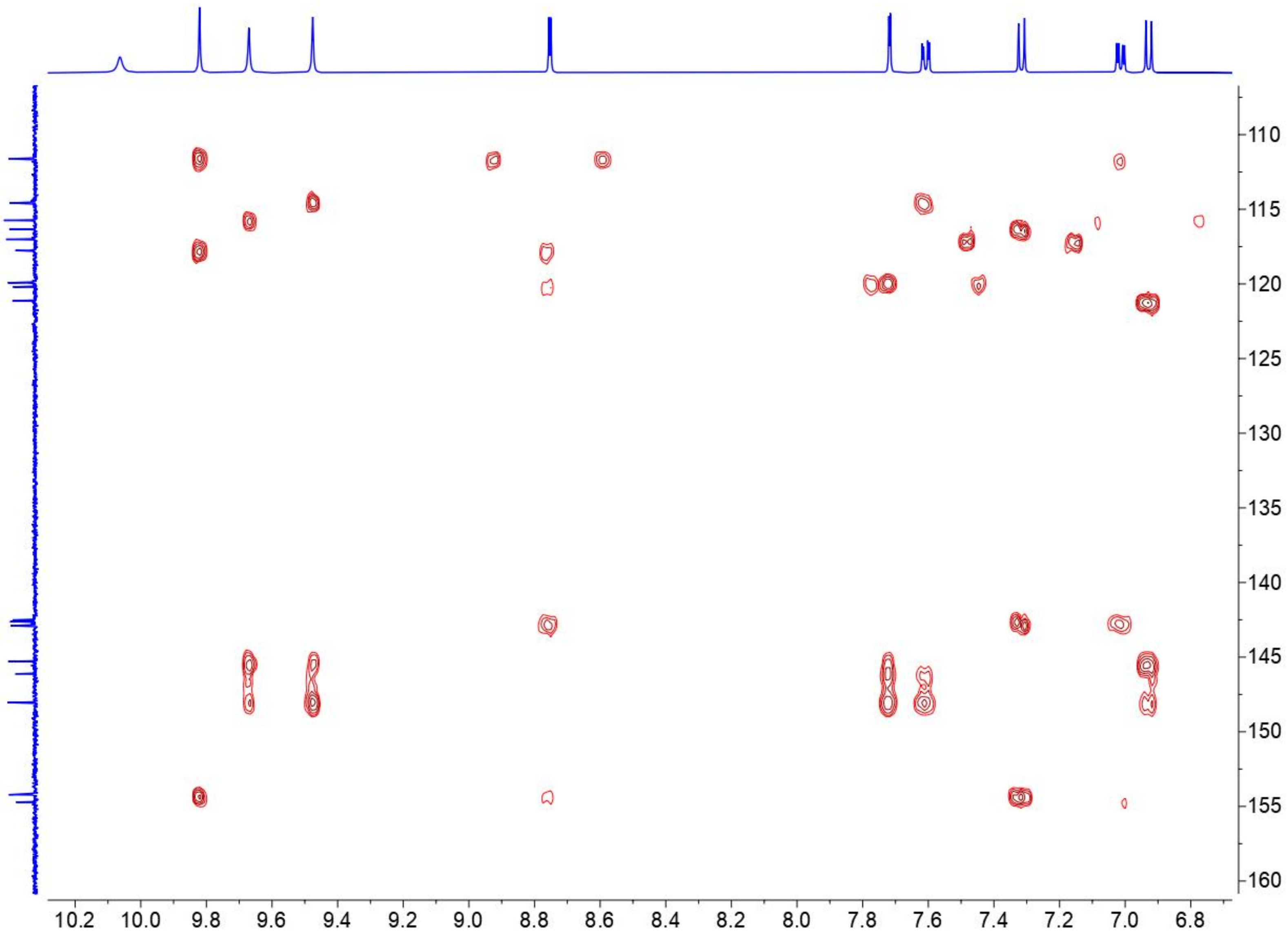

Figure S14. HMBC spectrum of compound **3**

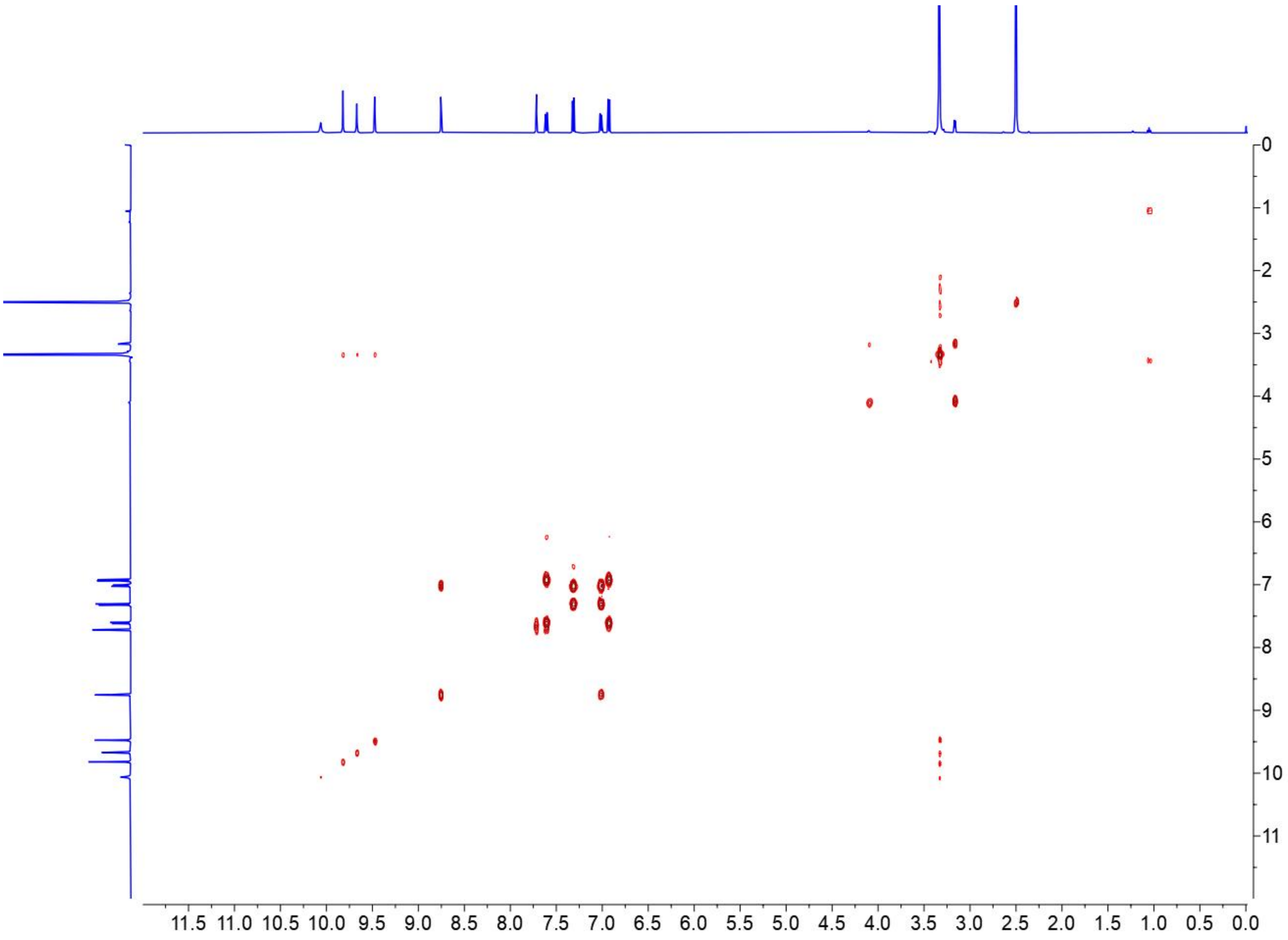

Figure S15. ^{1}H-^{1}H COSY spectrum of compound **3**

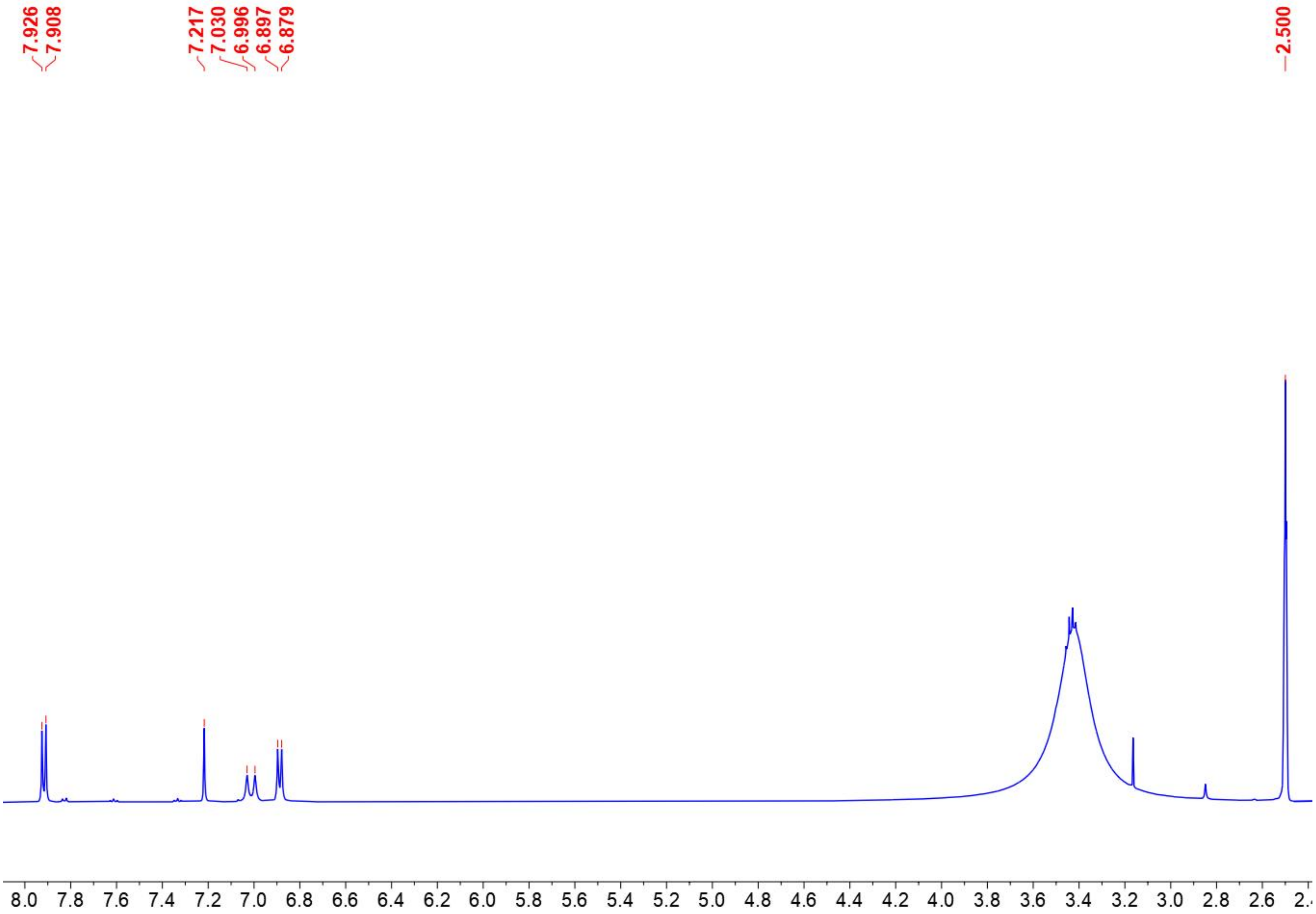

Figure S16. ^{1}H-NMR spectrum of compound **4**

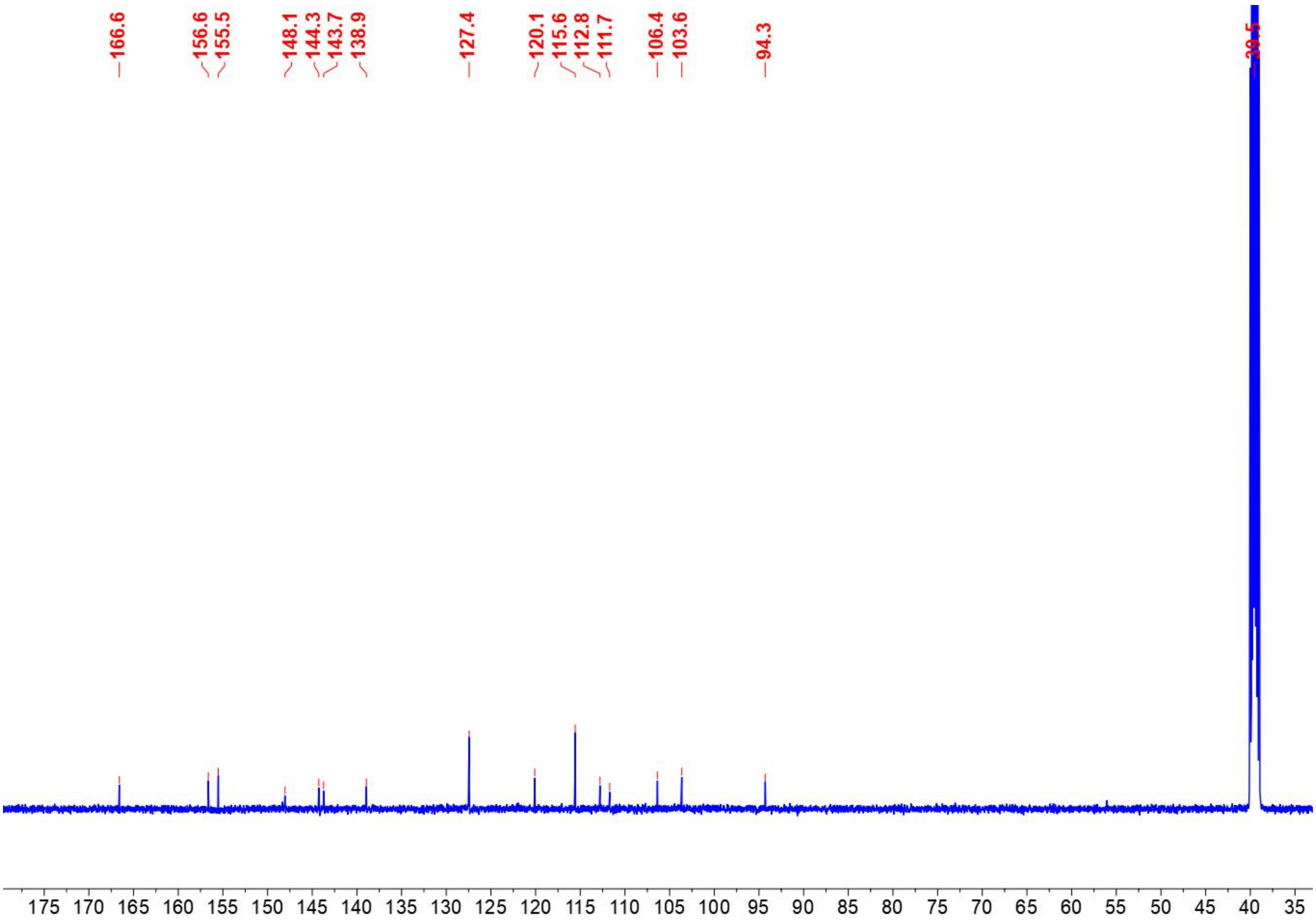

Figure S17. ^{13}C-NMR spectrum of compound **4**

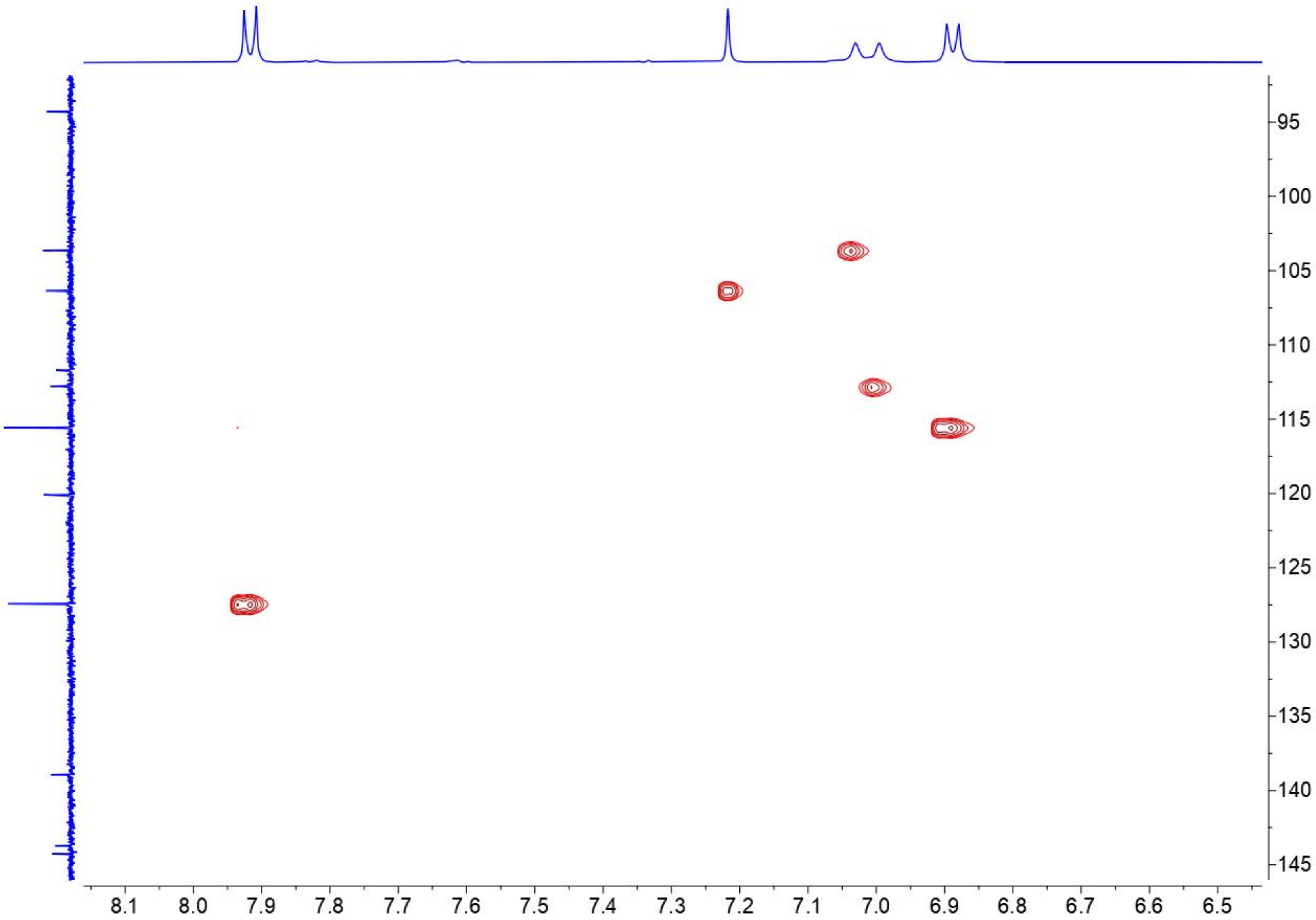

Figure S18. HSQC spectrum of compound **4**

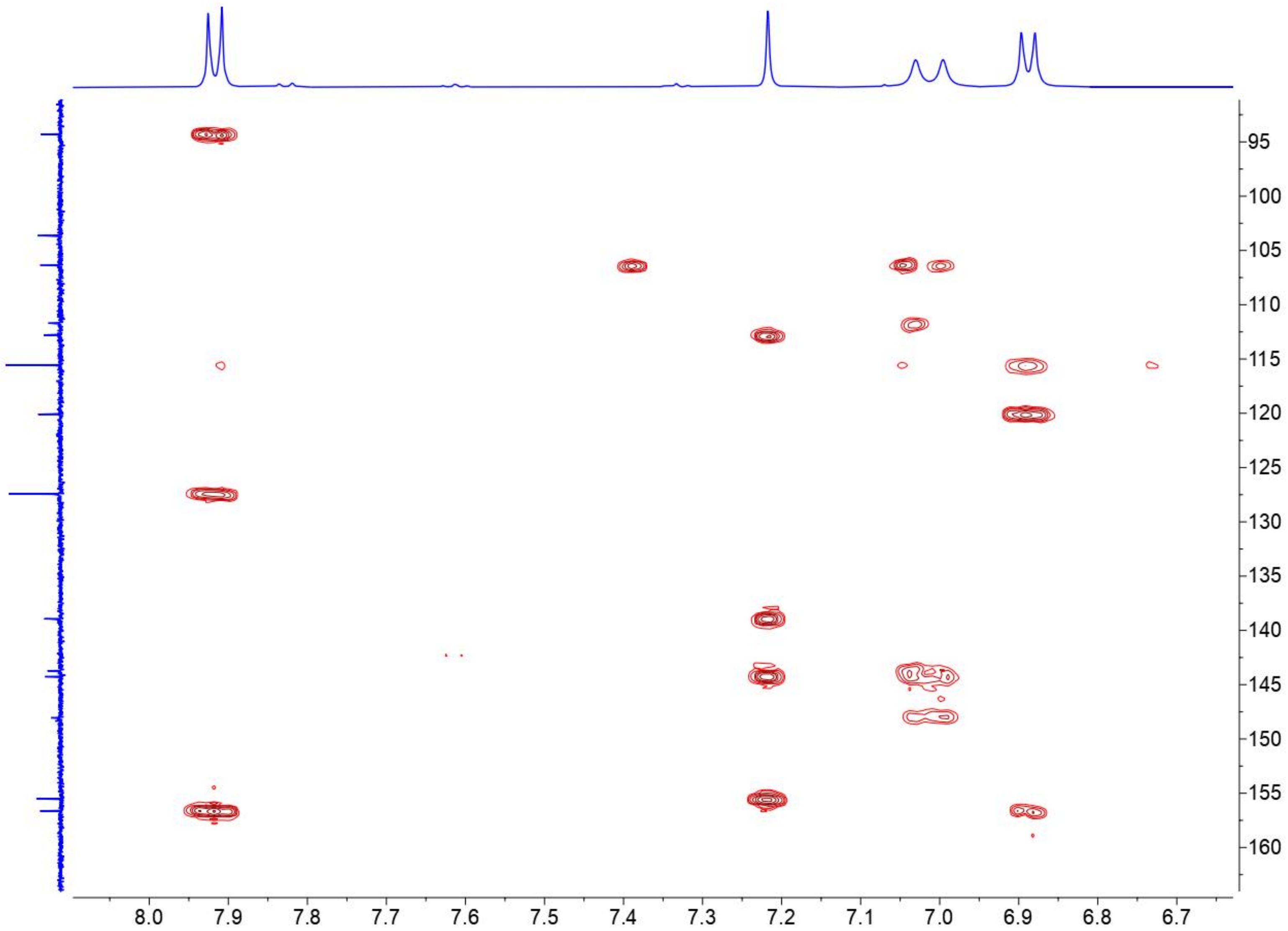

Figure S19. HMBC spectrum of compound **4**

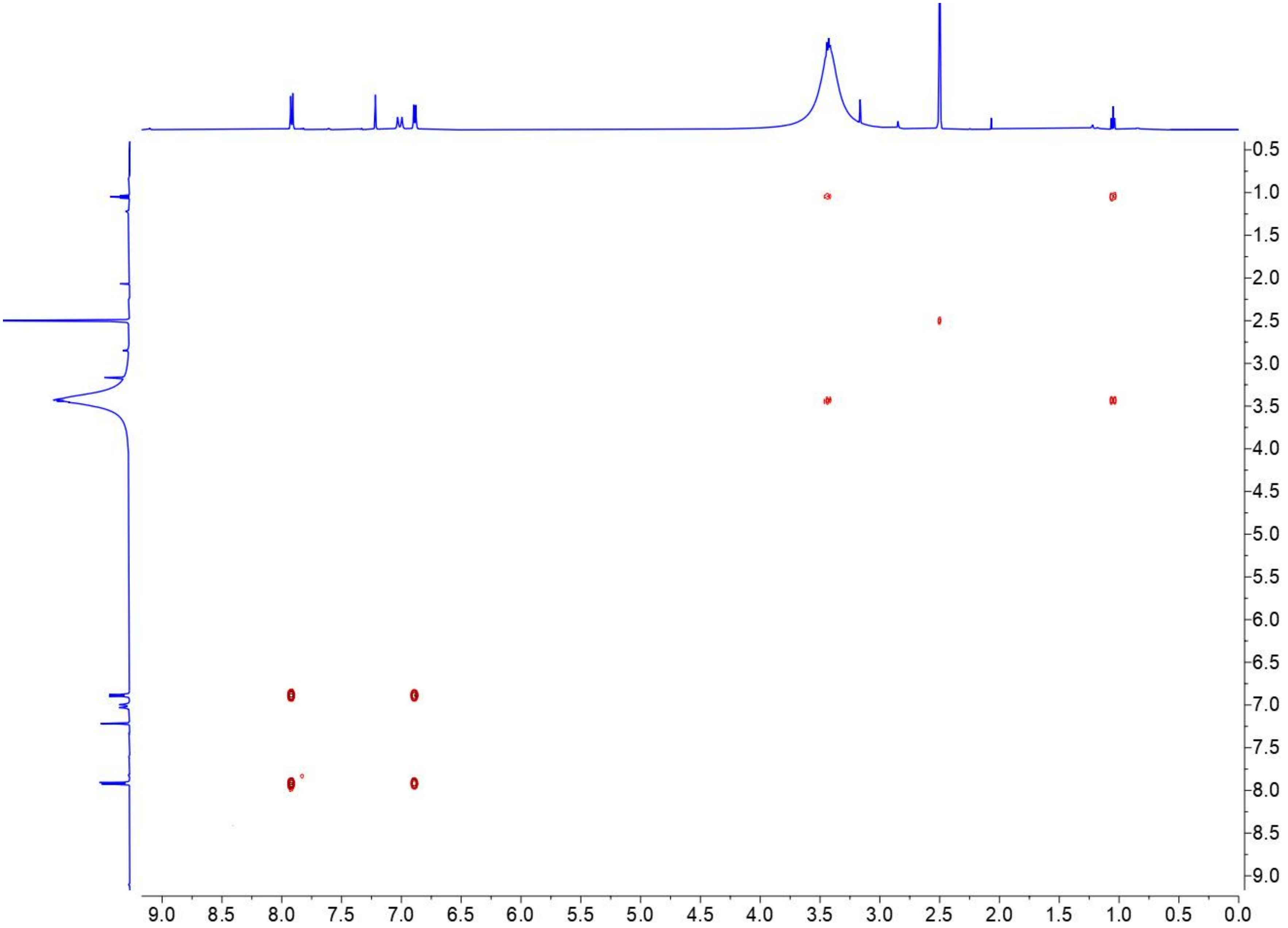

Figure S20. ^1H-^1H COSY spectrum of compound **4**

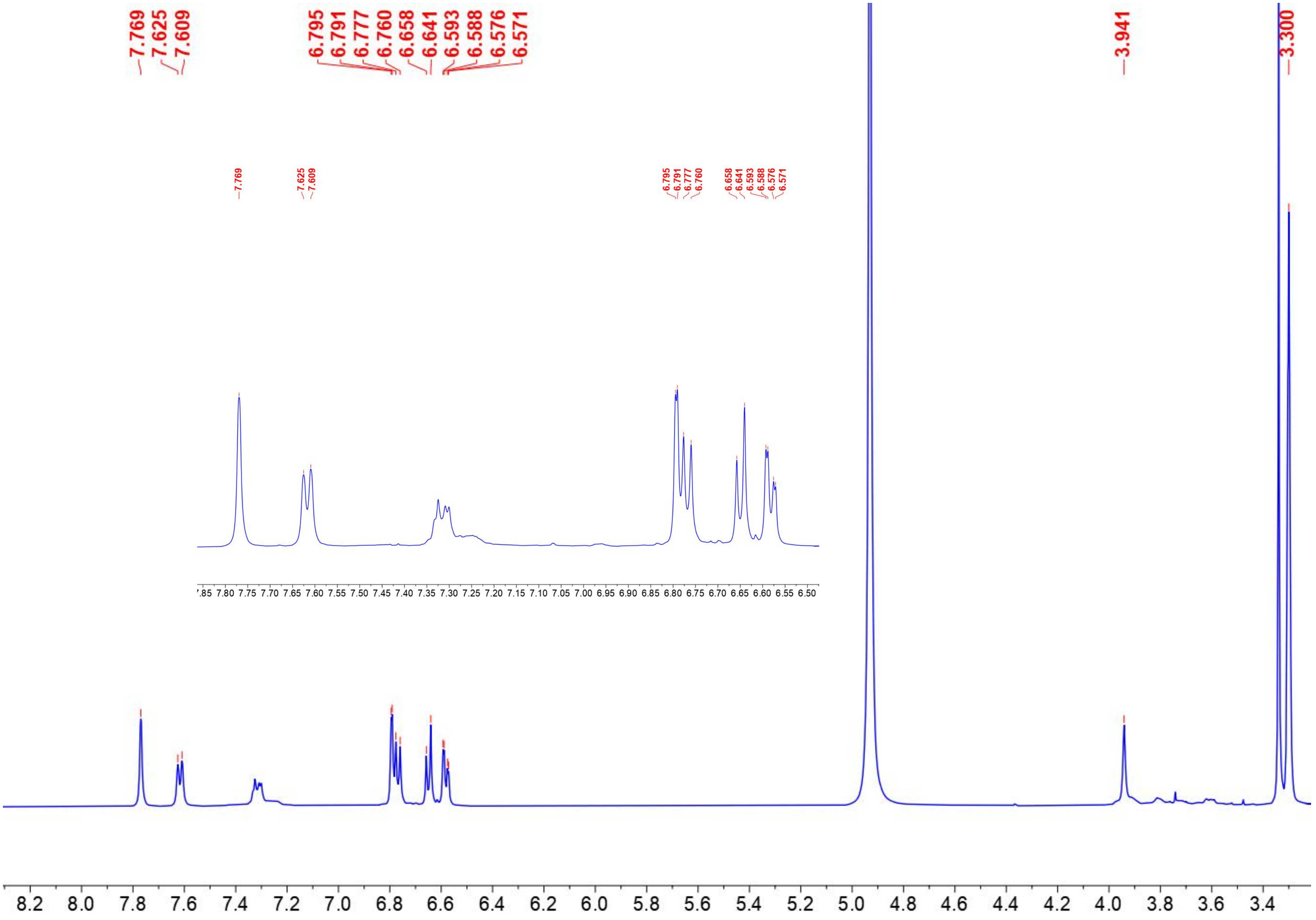

Figure S21. ^{1}H-NMR spectrum of compound **5**

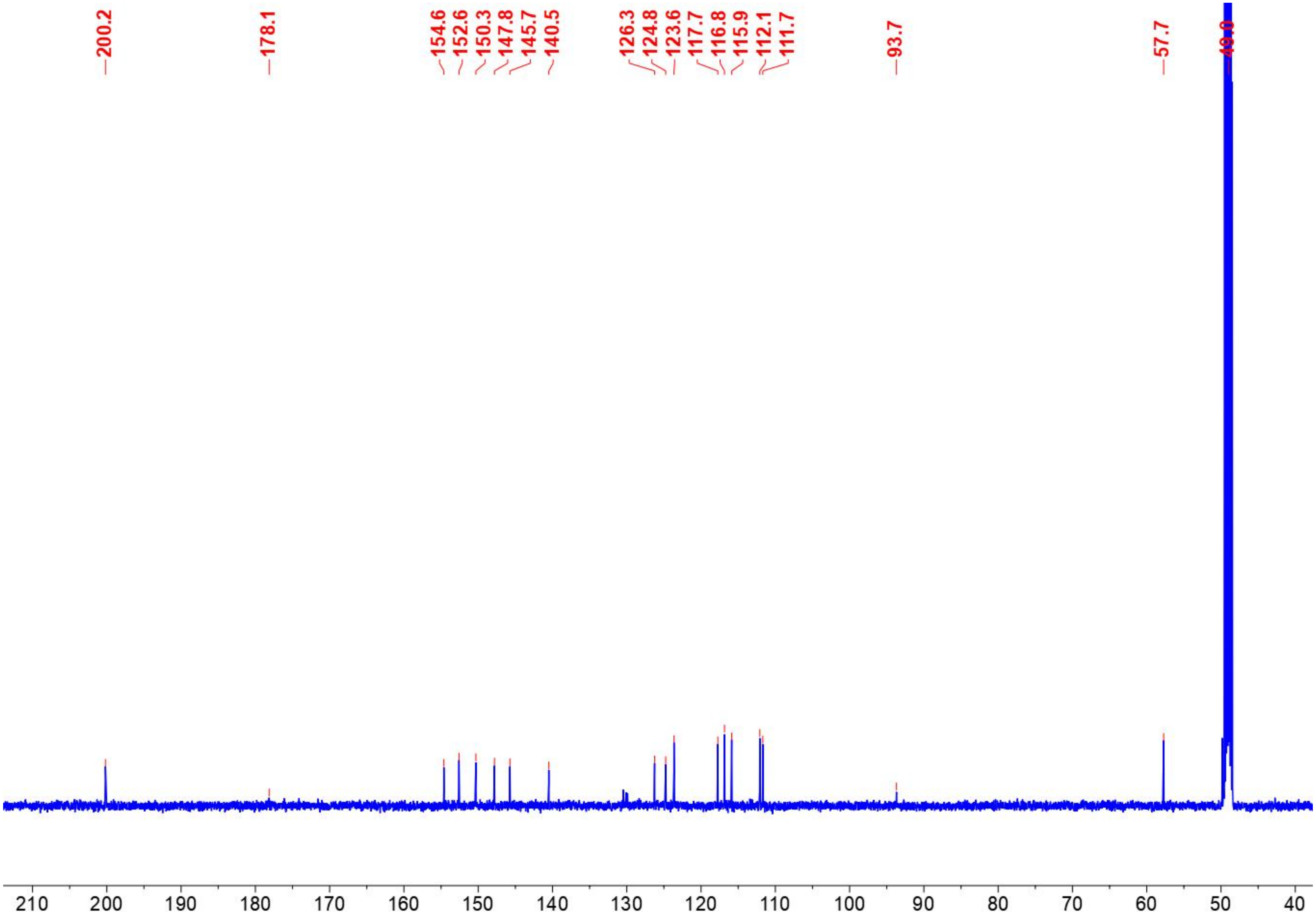

Figure S22. ^{13}C-NMR spectrum of compound **5**

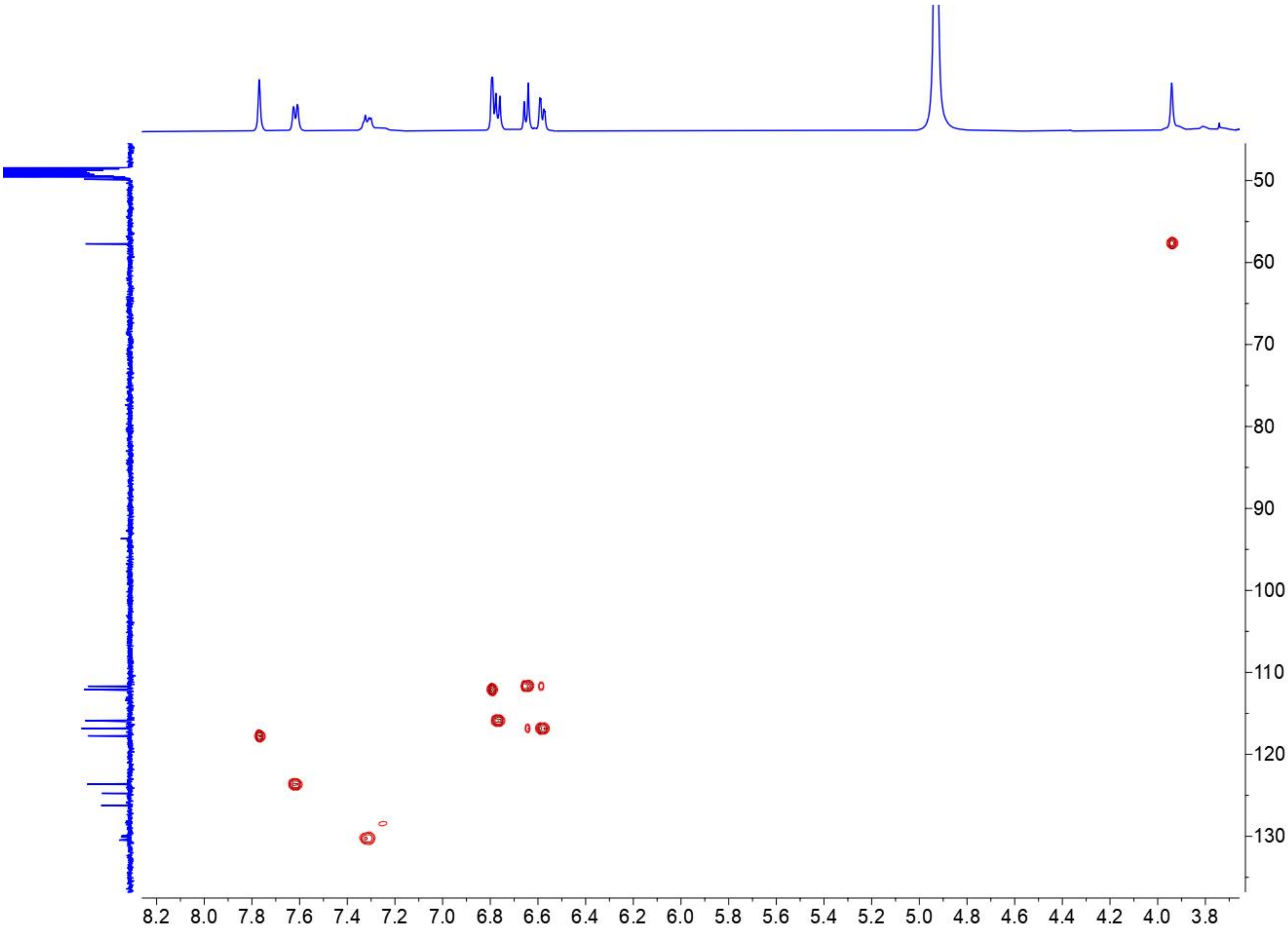

Figure S23. HSQC spectrum of compound **5**

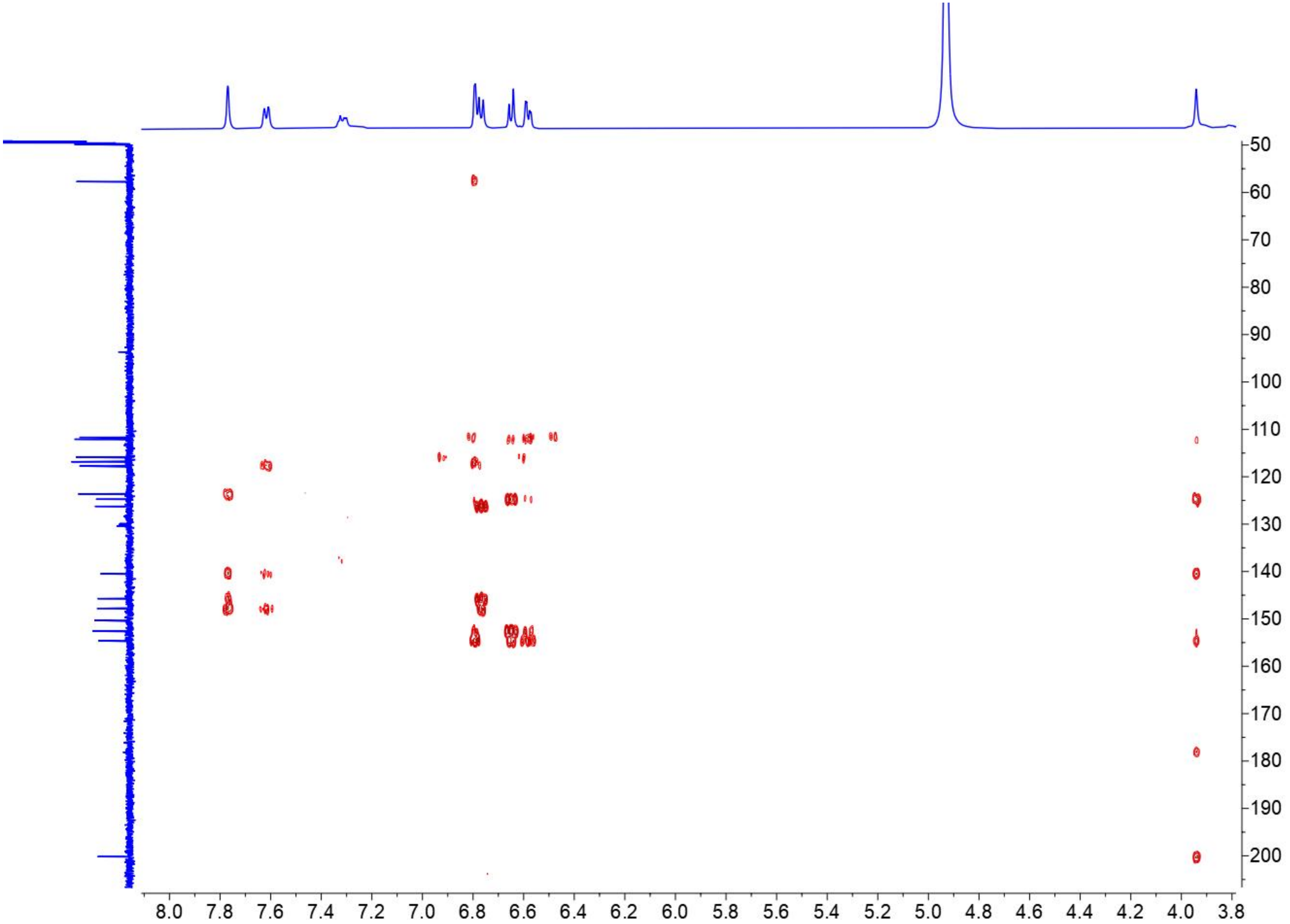

Figure S24. HMBC spectrum of compound **5**

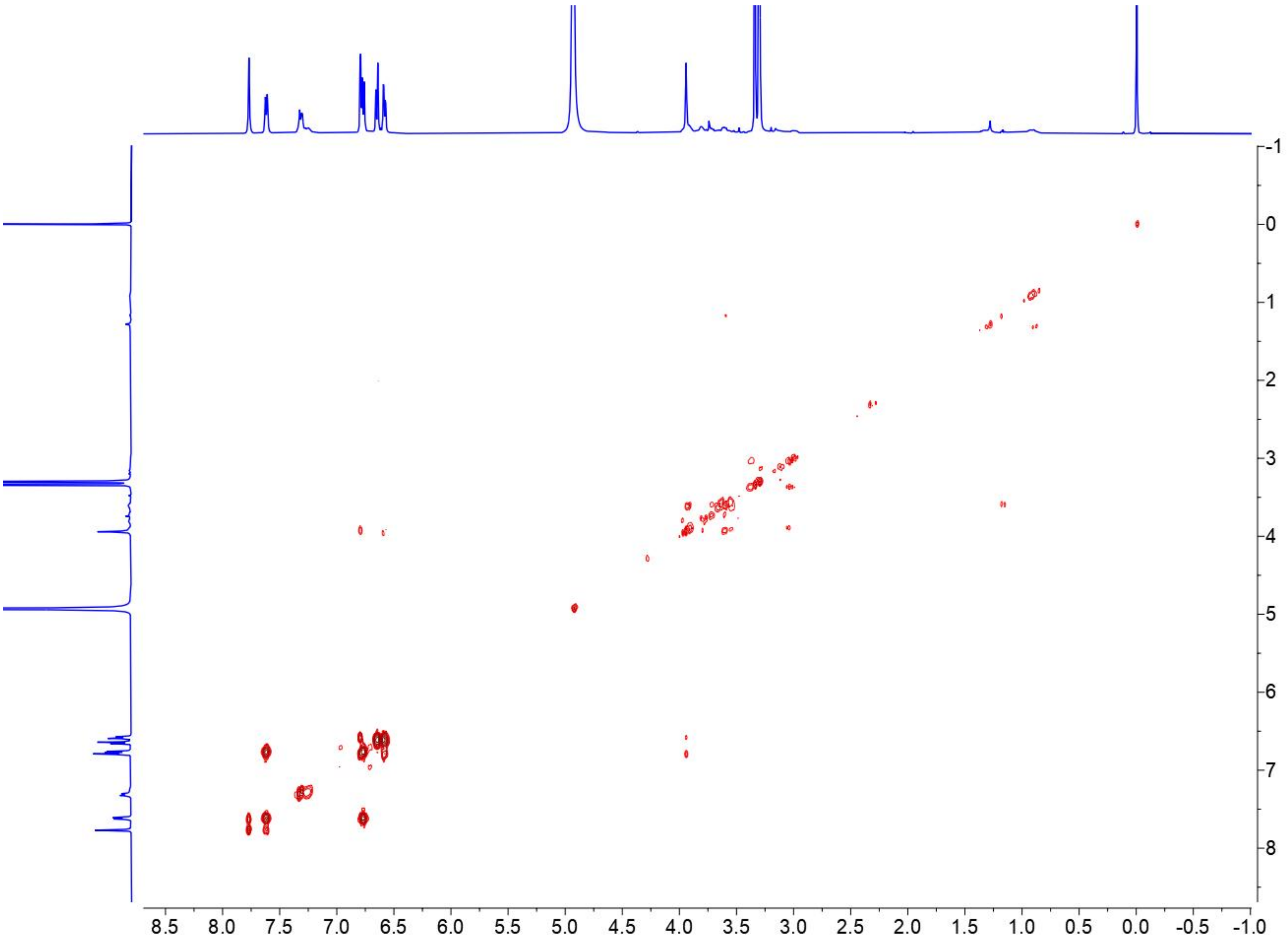

Figure S25. ^1H-^1H COSY spectrum of compound **5**

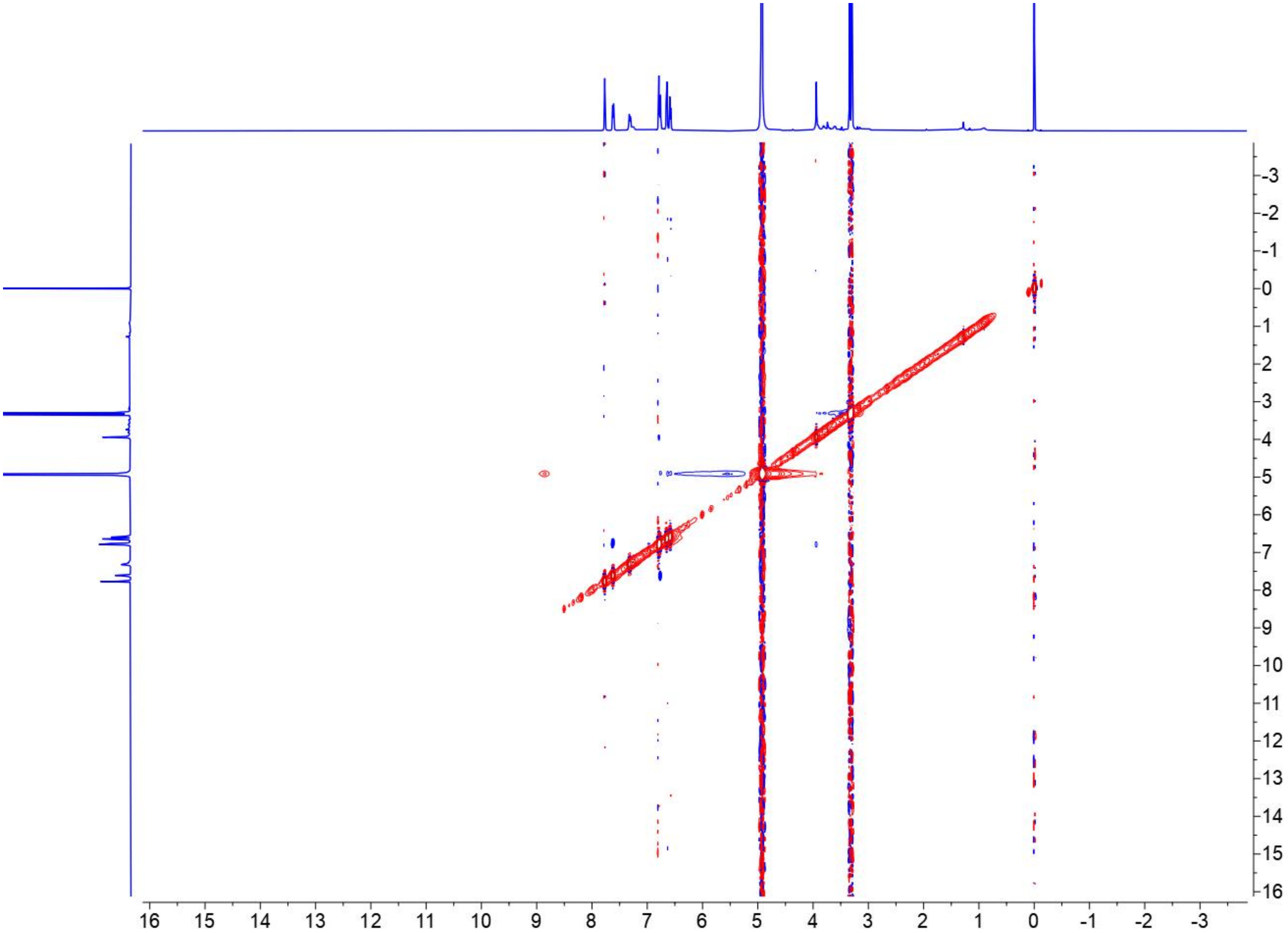

Figure S26. NOESY spectrum of compound **5**

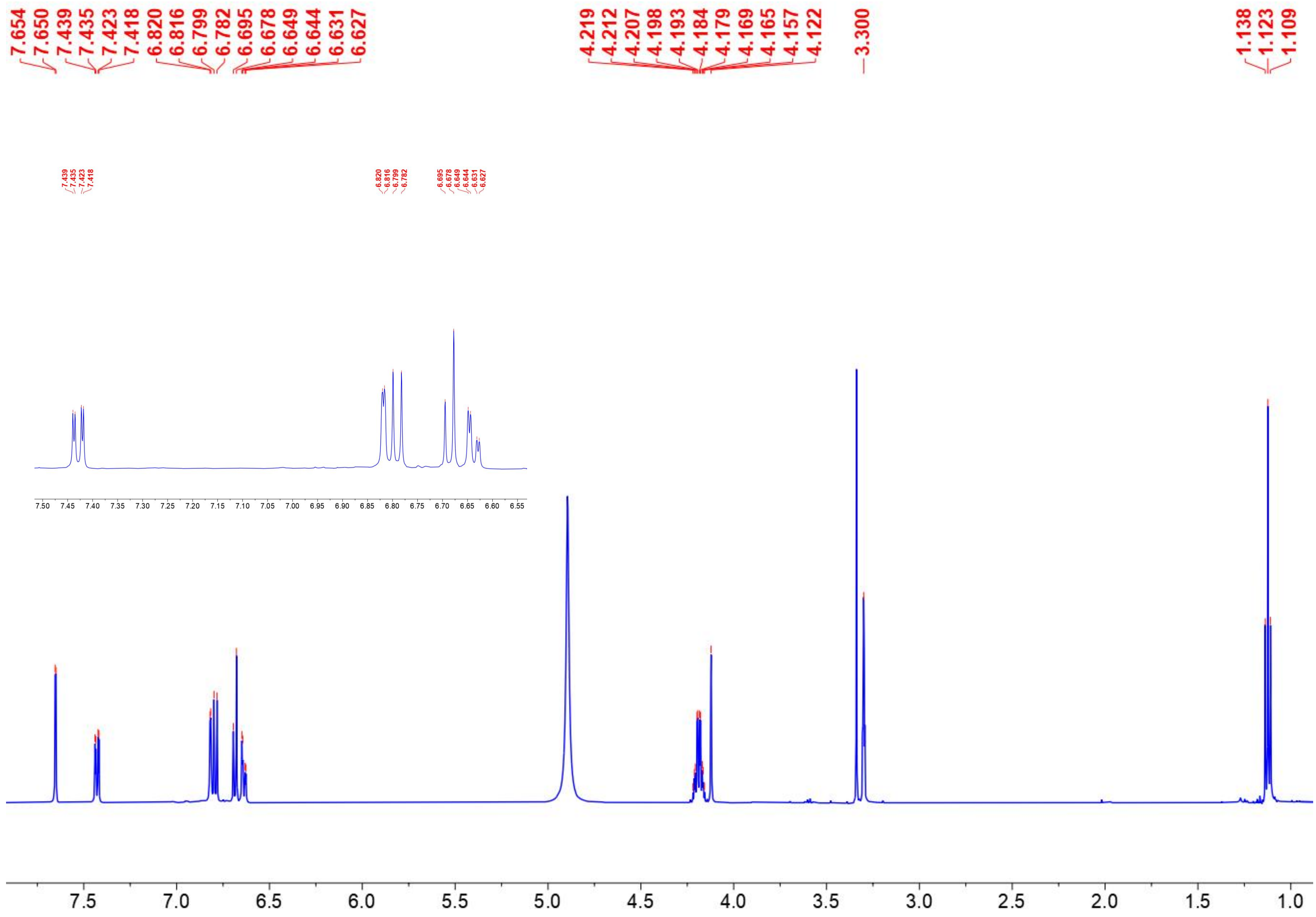
7.654
7.650
7.439
7.435
7.423
7.418
6.820
6.816
6.799
6.782
6.695
6.678
6.649
6.644
6.631
6.627
4.219
4.212
4.207
4.198
4.193
4.184
4.179
4.169
4.165
4.157
4.122
3.300
1.138
1.123
1.109
7.5
7.0
6.5
6.0
5.5
5.0
4.5
4.0
3.5
3.0
2.5
2.0
1.5
1.0

Figure S27. ^{1}H-NMR spectrum of compound **6**

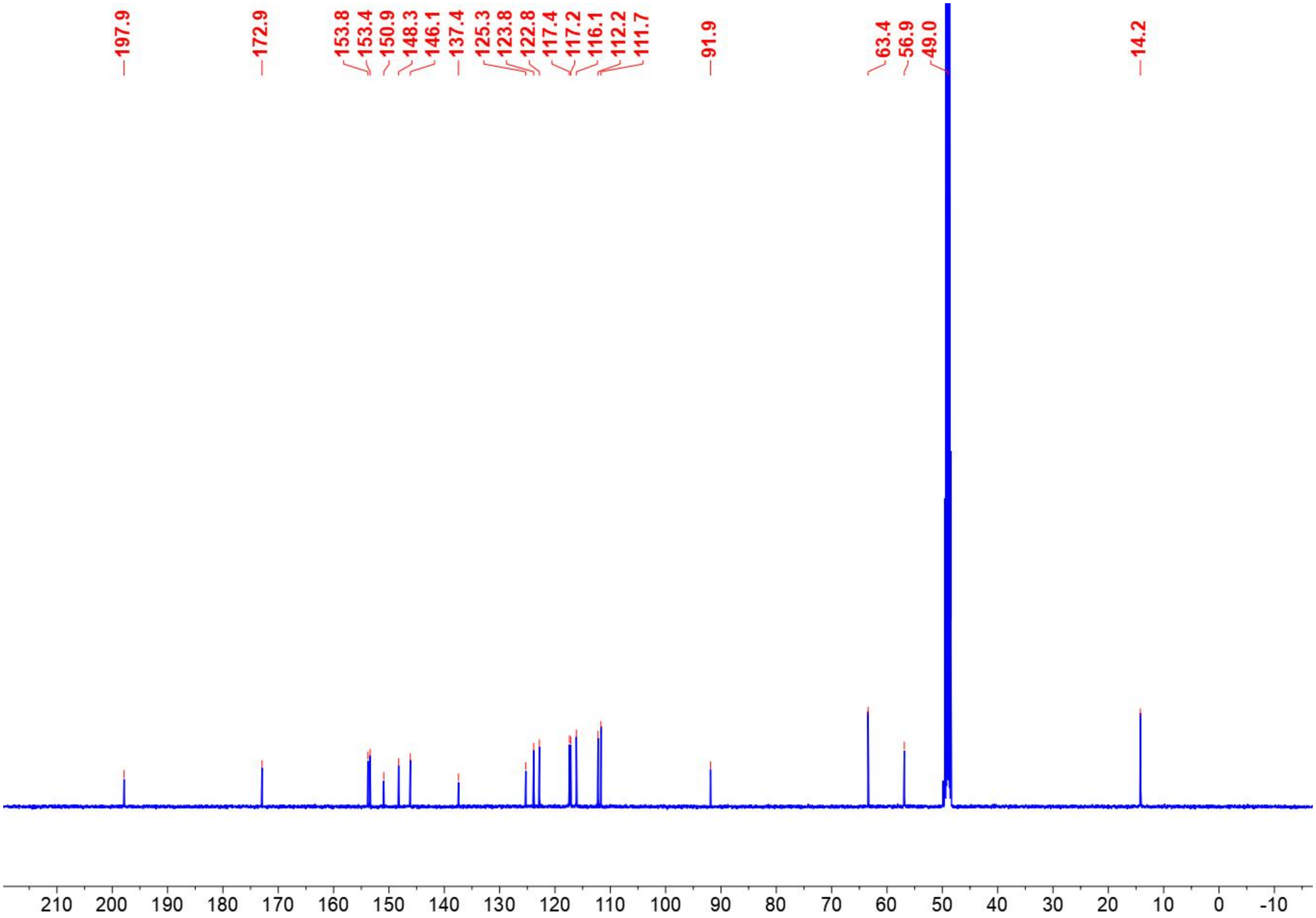

Figure S28. ^{13}C-NMR spectrum of compound **6**

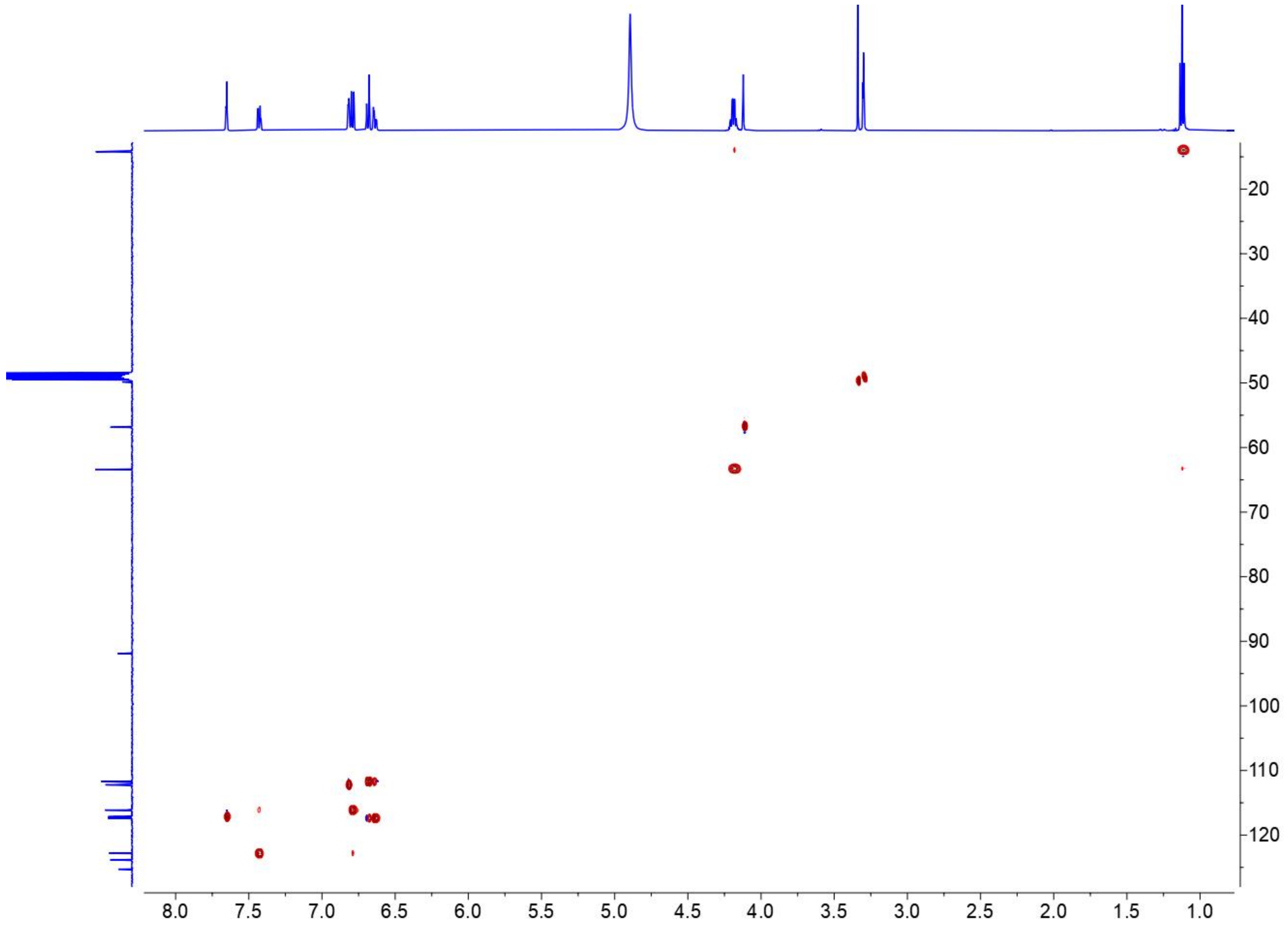
20
30
40
50
60
70
80
90
100
110
120
8.0
7.5
7.0
6.5
6.0
5.5
5.0
4.5
4.0
3.5
3.0
2.5
2.0
1.5
1.0

Figure S29. HSQC spectrum of compound **6**

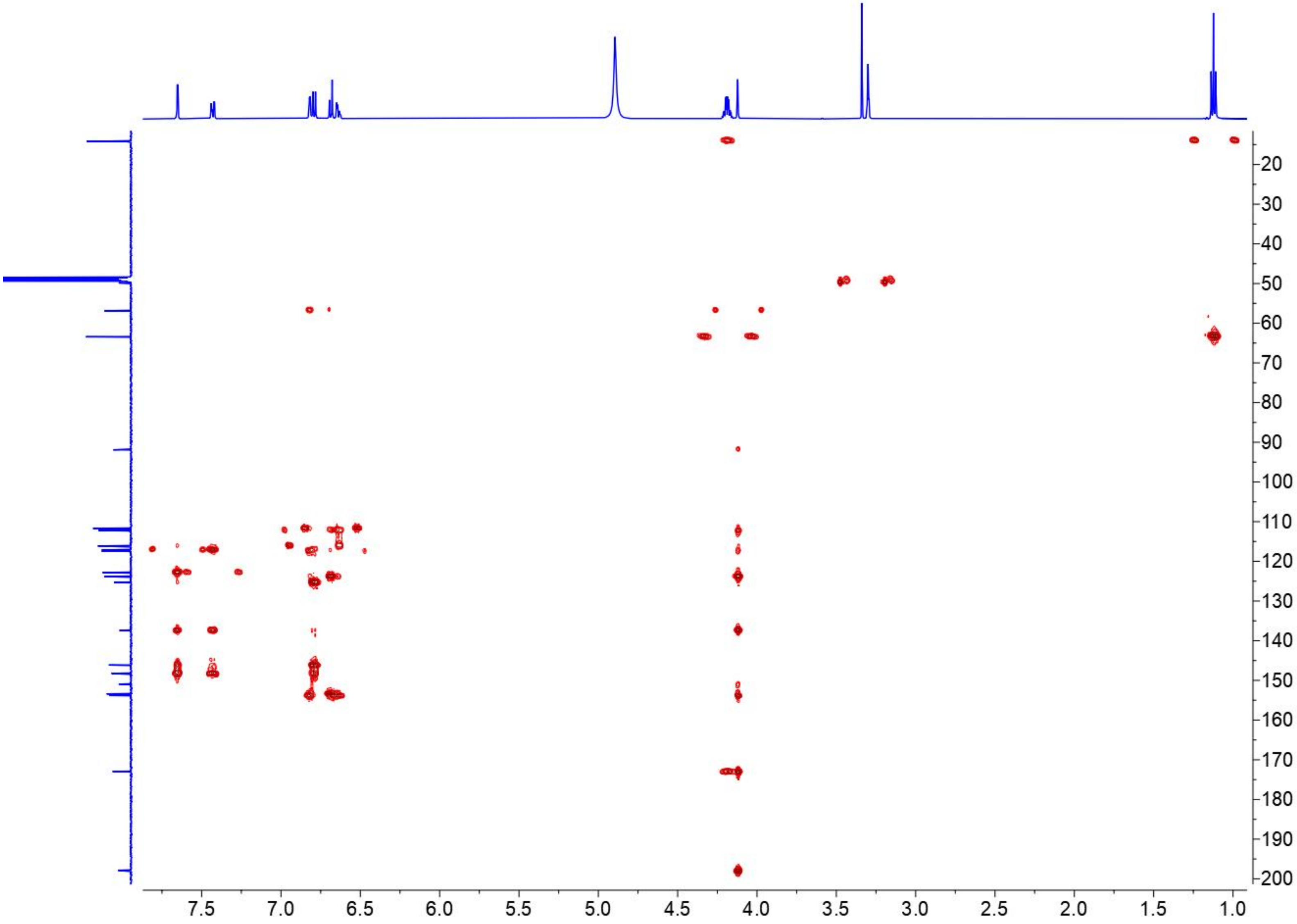

Figure S30. HMBC spectrum of compound **6**

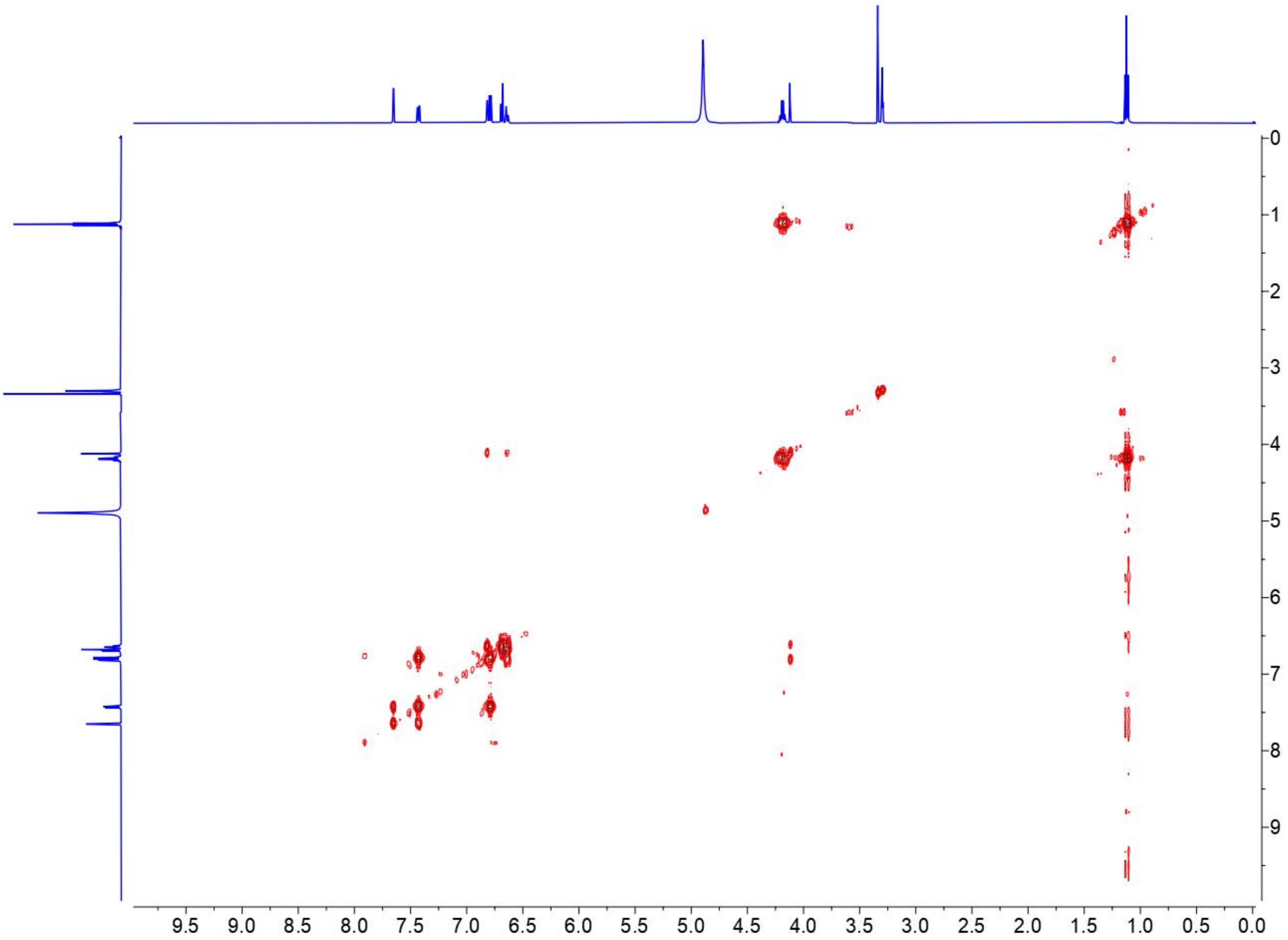

Figure S31. ^{1}H-^{1}H COSY spectrum of compound **6**

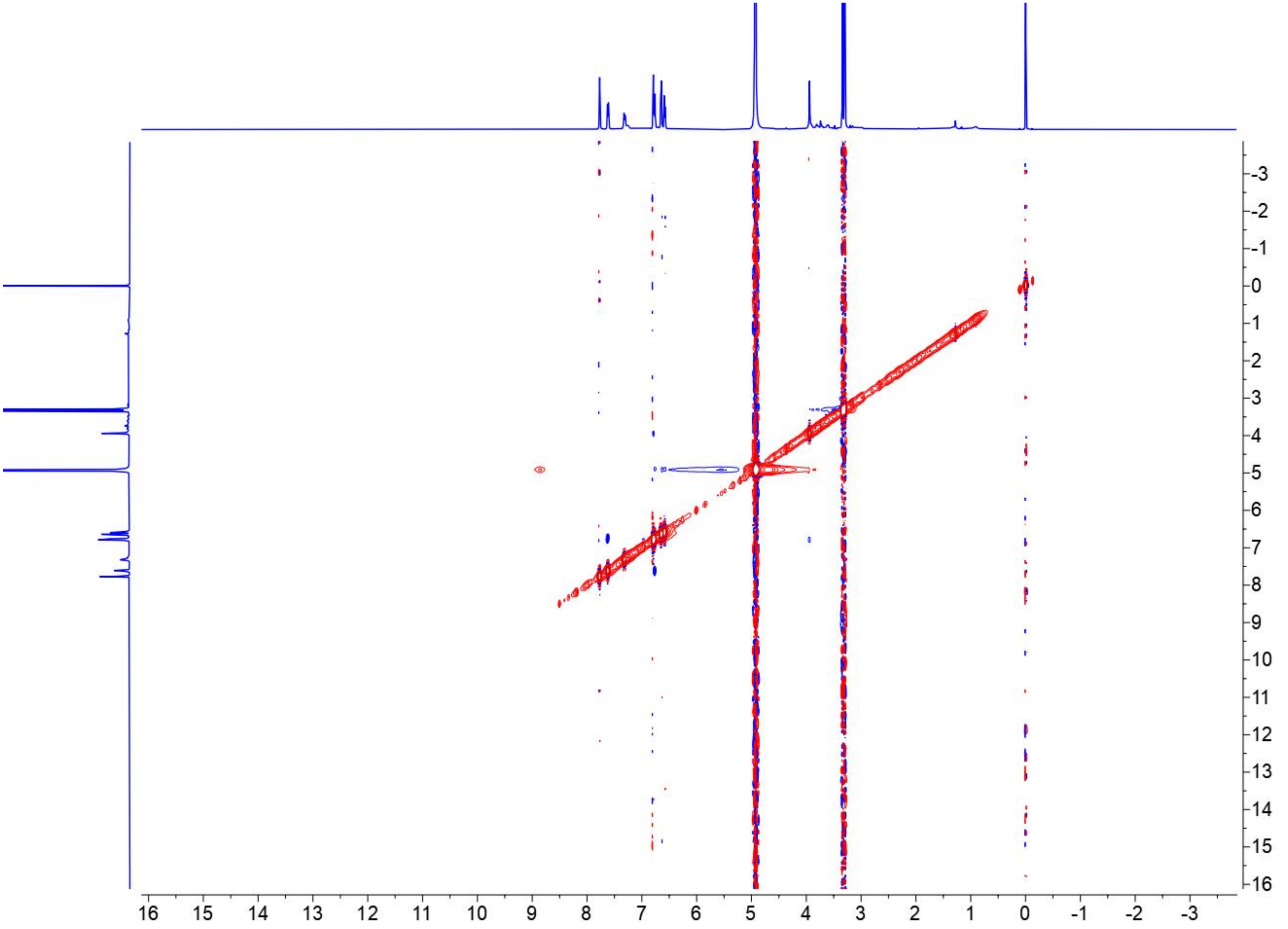

Figure S32. NOESY spectrum of compound **6**

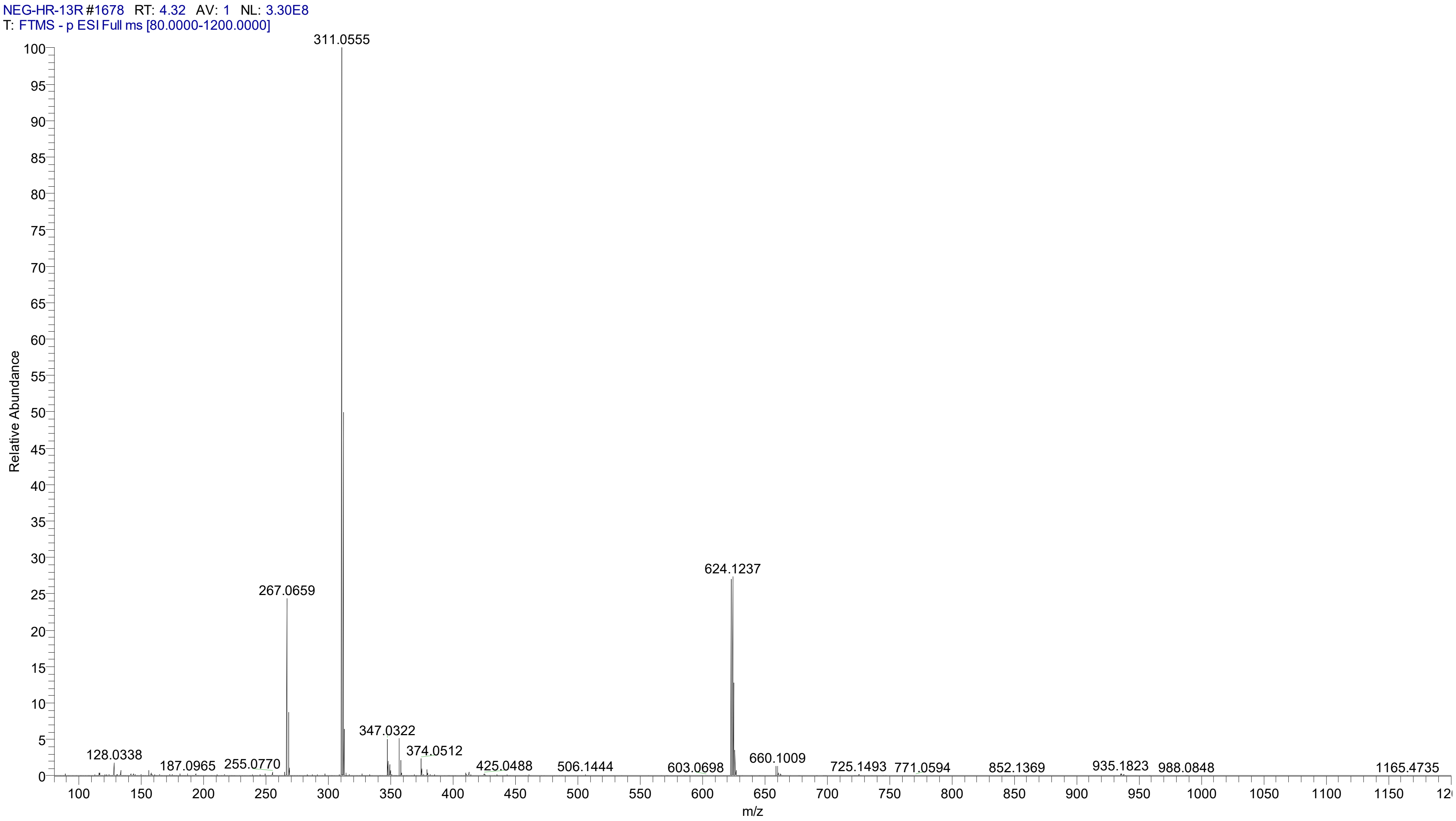

Figure S33. HR-ESI-MS of compound **1**

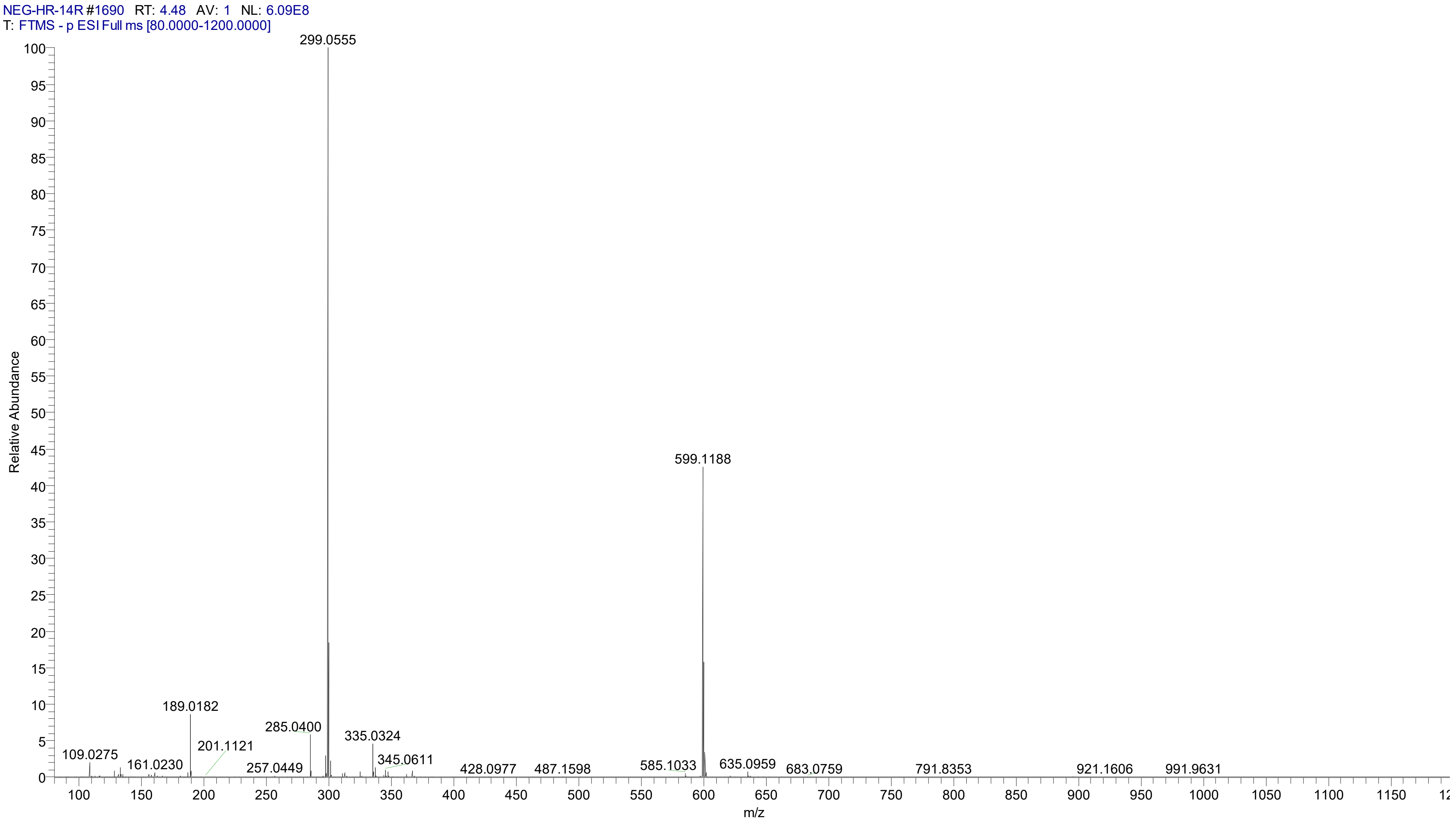

Figure S34. HR-ESI-MS of compound **2**

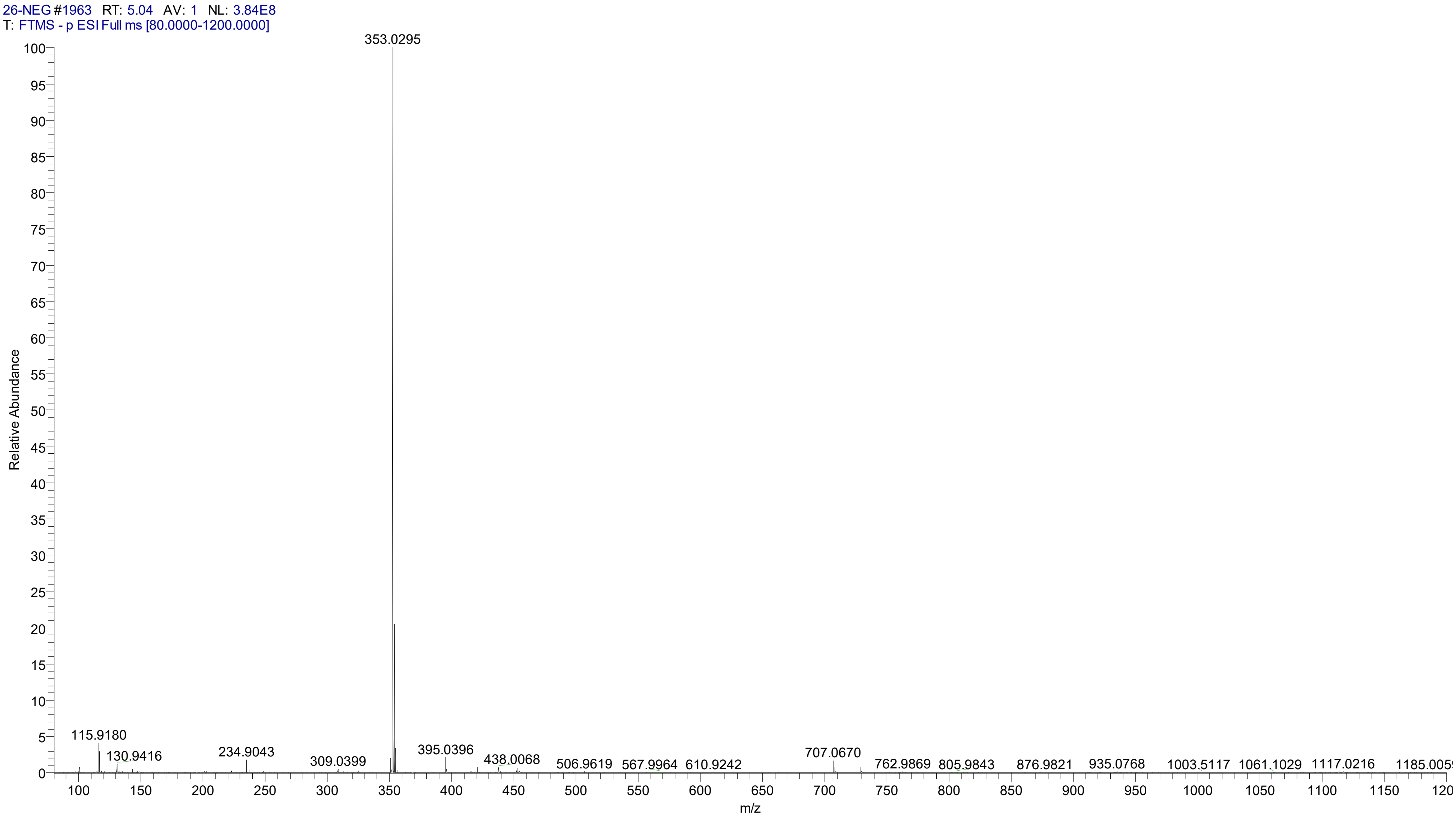

Figure S35. HR-ESI-MS of compound **3**

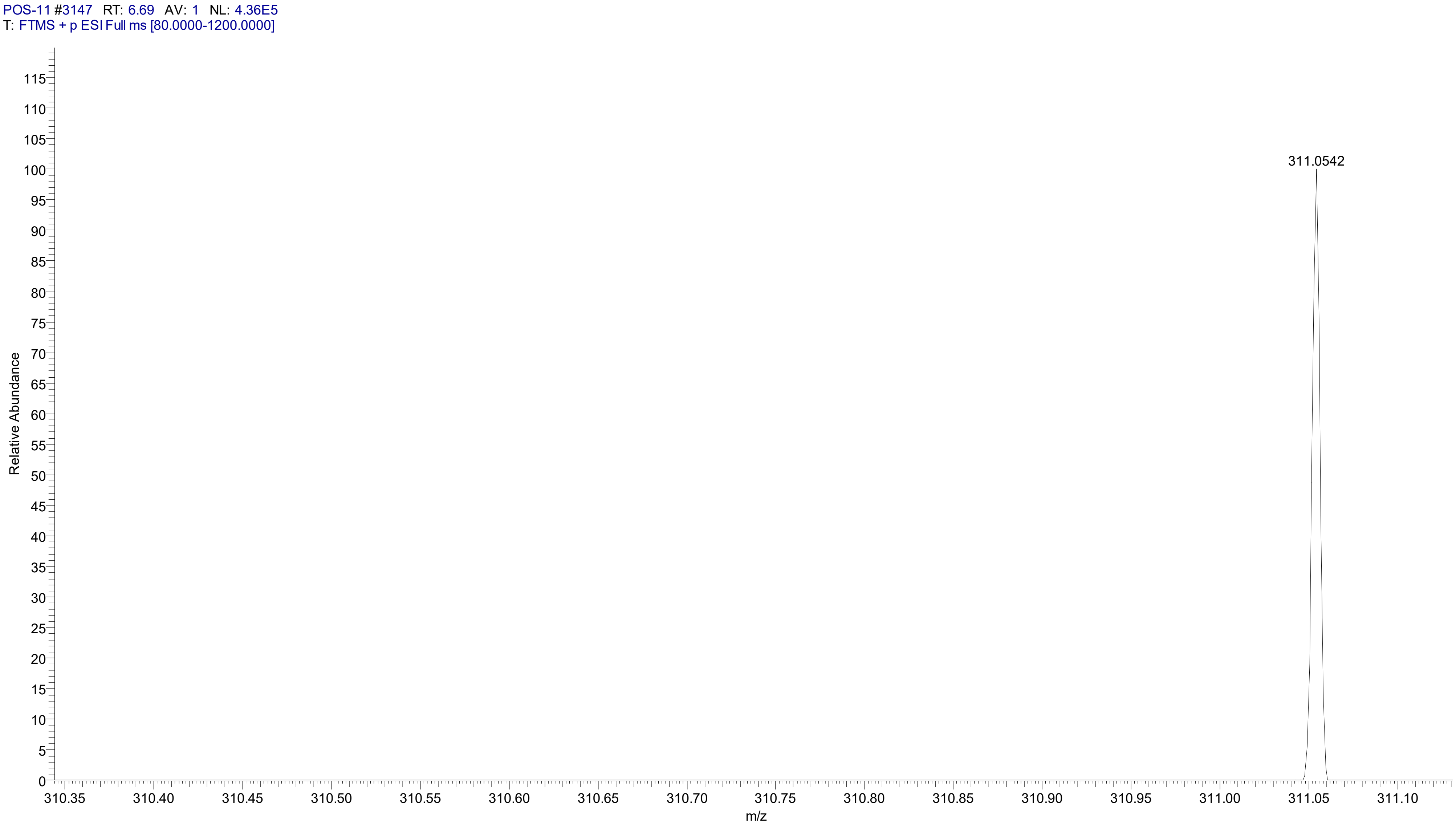

Figure S36. HR-ESI-MS of compound **4**

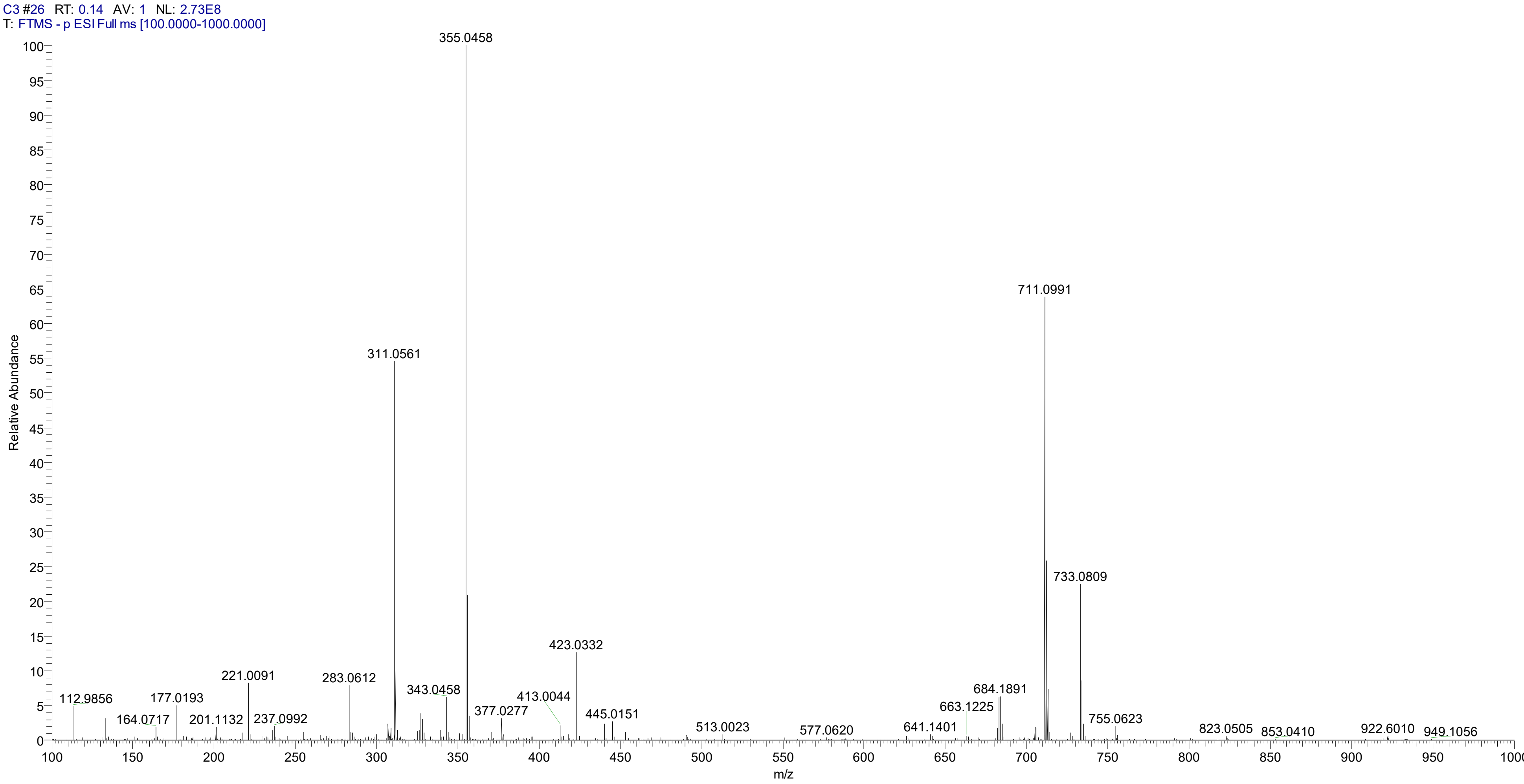

Figure S37. HR-ESI-MS of compound **5**

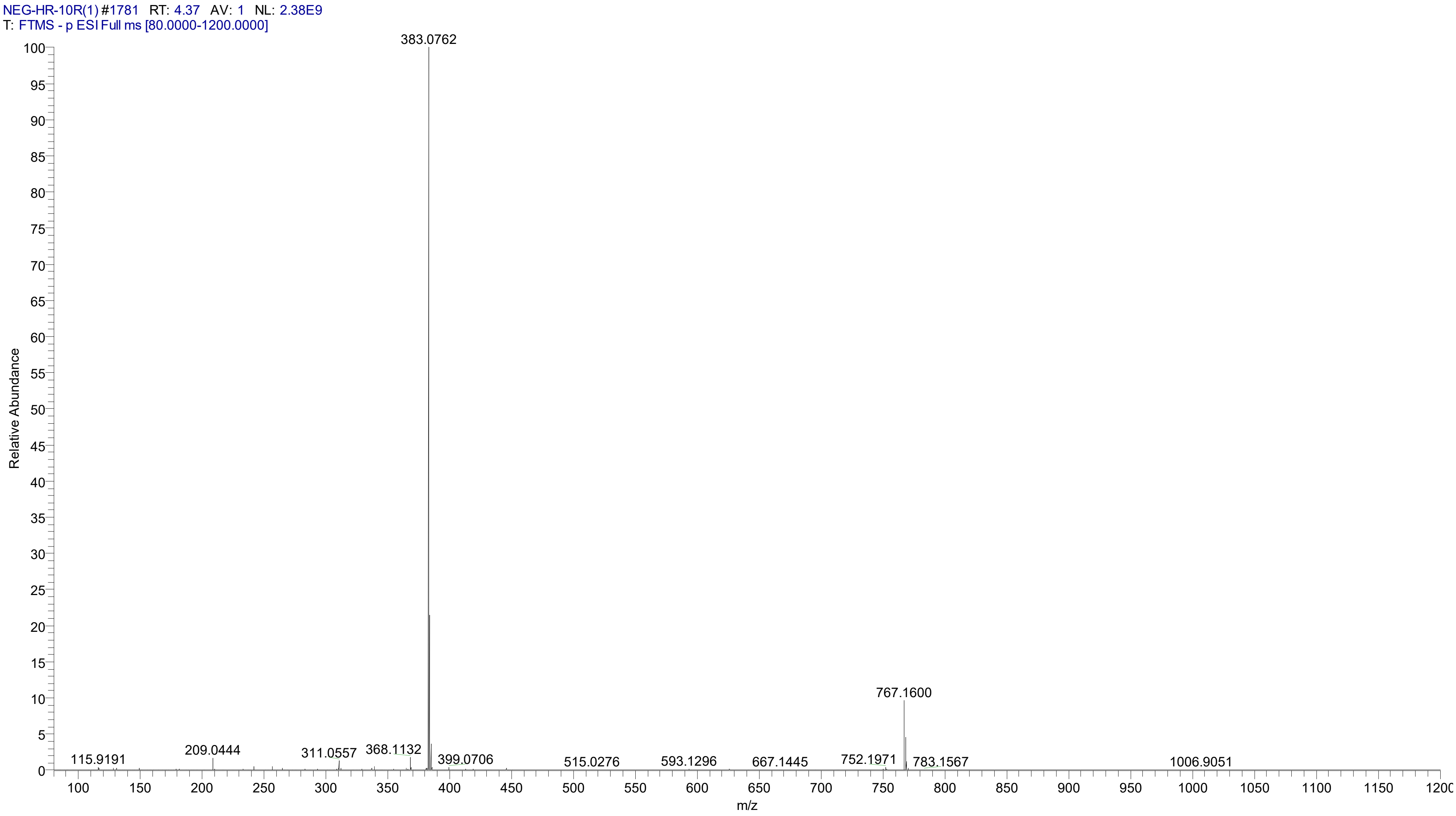

Figure S38. HR-ESI-MS of compound **6**
